# Nanoporous graphene-based thin-film microelectrodes for in vivo high-resolution neural recording and stimulation

**DOI:** 10.1038/s41565-023-01570-5

**Published:** 2024-01-11

**Authors:** Damià Viana, Steven T. Walston, Eduard Masvidal-Codina, Xavi Illa, Bruno Rodríguez-Meana, Jaume del Valle, Andrew Hayward, Abbie Dodd, Thomas Loret, Elisabet Prats-Alfonso, Natàlia de la Oliva, Marie Palma, Elena del Corro, María del Pilar Bernicola, Elisa Rodríguez-Lucas, Thomas Gener, Jose Manuel de la Cruz, Miguel Torres-Miranda, Fikret Taygun Duvan, Nicola Ria, Justin Sperling, Sara Martí-Sánchez, Maria Chiara Spadaro, Clément Hébert, Sinead Savage, Jordi Arbiol, Anton Guimerà-Brunet, M. Victoria Puig, Blaise Yvert, Xavier Navarro, Kostas Kostarelos, Jose A. Garrido

**Affiliations:** 1 Catalan Institute of Nanoscience and Nanotechnology (ICN2), CSIC and BIST, Campus UAB, Barcelona, Spain; 2 Institut de Microelectrònica de Barcelona, IMB-CNM (CSIC), Campus UAB, Bellaterra, Spain; 3 Centro de Investigación Biomédica en Red de Bioingeniería, Biomateriales y Nanomedicina, Instituto de Salud Carlos III, Madrid, Spain; 4 Institute of Neurosciences, Department of Cell Biology, Physiology and Immunology, Centro de Investigación Biomédica en Red sobre Enfermedades Neurodegenerativas (CIBERNED), Universitat Autònoma de Barcelona, Barcelona, Spain; 5 Secció de Fisiologia, Department de Bioquímica i Fisiologia, Facultat de Farmàcia i Ciències de l’Alimentació, Institut de Neurociències, Universitat de Barcelona, Barcelona, Spain; 6 Nanomedicine Lab, National Graphene Institute and Faculty of Biology, Medicine & Health, Manchester, UK; 7 Univ. Grenoble Alpes, Inserm, U1216, Grenoble Institut Neurosciences, Grenoble, France; 8 Hospital del Mar Research Institute, Barcelona, Spain; 9 ICREA, Barcelona, Spain

**Keywords:** Bionanoelectronics, Biomaterials

## Abstract

One of the critical factors determining the performance of neural interfaces is the electrode material used to establish electrical communication with the neural tissue, which needs to meet strict electrical, electrochemical, mechanical, biological and microfabrication compatibility requirements. This work presents a nanoporous graphene-based thin-film technology and its engineering to form flexible neural interfaces. The developed technology allows the fabrication of small microelectrodes (25 µm diameter) while achieving low impedance (∼25 kΩ) and high charge injection (3–5 mC cm^−^^2^). In vivo brain recording performance assessed in rodents reveals high-fidelity recordings (signal-to-noise ratio >10 dB for local field potentials), while stimulation performance assessed with an intrafascicular implant demonstrates low current thresholds (<100 µA) and high selectivity (>0.8) for activating subsets of axons within the rat sciatic nerve innervating tibialis anterior and plantar interosseous muscles. Furthermore, the tissue biocompatibility of the devices was validated by chronic epicortical (12 week) and intraneural (8 week) implantation. This work describes a graphene-based thin-film microelectrode technology and demonstrates its potential for high-precision and high-resolution neural interfacing.

## Abstract (web summary)

Bidirectional neural interface electronic devices offer therapeutic options. Here, the authors present wafer-scale fabrication of flexible nanoporous graphene-based implantable microelectrode arrays with low impedance and high charge injection for in vivo brain recording and nerve stimulation.

## Main

Neural interface medical devices offer therapeutic options to patients suffering from certain neurological disorders and neural impairments (for example, Parkinson’s disease^1^, deafness^2^ or amputations^3^)^4,5^. To broaden the range of clinical uses of neural interfaces, improvements in efficacy are needed so that the treatment benefit outweighs the associated risks^6–9^. Current clinical technology mostly consists of devices that either electrically record or stimulate the nervous system using millimetre-scale metallic electrodes. To improve the interface with the nervous system, multiple lines of research suggest that the electrode dimensions should be miniaturized to the micrometre-scale^3,5,9–11^, allowing neural recordings to be captured at a higher spatial resolution, potentially resulting in improved neural signal decoding^8,12,13^. Additionally, the reduced electrode size can improve stimulation focality and facilitate recapitulation of the natural neural activation patterns of healthy nervous tissue^9,14,15^.

Research on novel materials and electrode coatings has attempted improvements in neural recording and stimulation performance^16–19^. Aside from classically used noble metals, such as gold and platinum, nanoengineered metals^20^, metal oxides^16^, conducting polymers^21,22^ and carbon-based materials^23,24^, among others^16^, have been explored as alternative material options to engineer neural interfaces. Such research efforts are very much active today, particularly in the design of miniaturized, implantable neural interfaces for chronic use^25,26^.

Due to their unique combination of properties, graphene-related materials have emerged as attractive candidates for electrode fabrication in bidirectional neural interfaces^10,27,28^. Graphene electrodes offer a capacitive interaction in aqueous media over a wide potential window coupled with mechanical flexibility^10,29^. Single-layer graphene microelectrodes have been used for neural interfacing applications, but the limited electrochemical performance of this carbon monolayer constrains the potential for miniaturization^27^. To improve performance, multilayer porous electrodes have been explored^28^ but their development has proven to be very challenging. This is mainly due to difficulties of obtaining high porosity, yet dense packing of the material layers and a high ion-accessible surface area with low ion transport resistance. Current achievements have lowered the impedance and increased the charge injection limit (CIL) of graphene-based electrodes^30^. However, to date only bulky porous electrodes of hundreds of micrometres of thickness have been demonstrated^31^, which limits the integration of the technology into dense arrays for use with anatomically congruent interfaces.

We describe here a graphene-based thin-film electrode material (Engineered Graphene for Neural Interface (EGNITE)) and a wafer-scale fabrication process of flexible microelectrode arrays for high spatial resolution neural recording and stimulation. EGNITE microelectrodes exhibit low impedance, high CIL and biologically relevant current pulse stimulation stability. EGNITE performance for bidirectional neural interfacing has been validated in rodents. Cortical recording studies confirm the ability to record spontaneous and evoked local field potentials and multiunit activity (MUA). Intraneural placement within the sciatic nerve made it possible to explore spatially precise stimulation for selective muscle activation. Additionally, chronic in vivo biocompatibility studies have been performed to assess tissue response to the implanted devices.

## Electrode material and array microfabrication

**Fig. 1:**
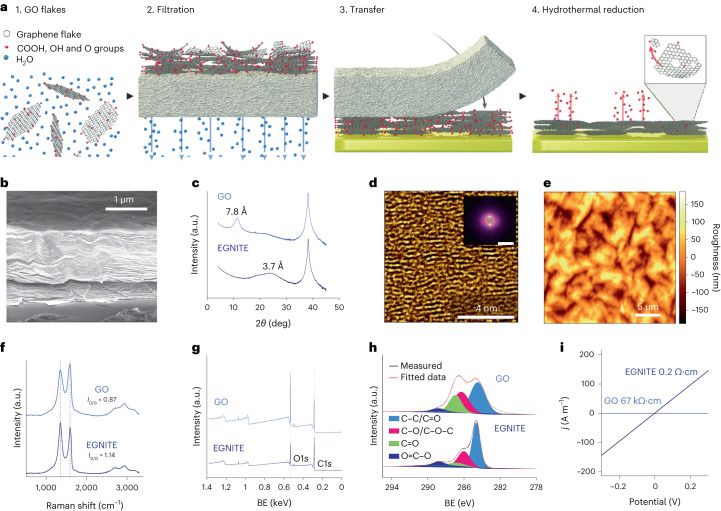
Preparation of nanoporous reduced GO thin films. **a**, Preparation of the porous reduced GO thin-film EGNITE. This consists of filtering a GO solution through a porous membrane (1, 2), transferring the deposited film of stacked GO flakes onto a conductive substrate (3) and the hydrothermal reduction of the ensemble, which turns the film highly porous and conductive (4). **b**, SEM micrograph of a cross section of the material. **c**, X-ray diffraction of GO and EGNITE, revealing the characteristic peaks corresponding to the parallel stacking of the GO and reduced GO flakes. **d**, HRTEM false-colour cross-sectional view of EGNITE. Inset: corresponding power spectrum showing two symmetric diffuse spots, indicating the preferred stacking direction in the material and slight fluctuation of the flakes’ interplanar distance. Scale bar, 0.1 nm. **e**, AFM image revealing roughness of the upper surface of the EGNITE film. **f**, Raman spectra of the GO and EGNITE. The ratio between D and G peaks increases after the hydrothermal treatment. **g**, XPS full spectrum. BE, binding energy. **h**, C1*s* peak of (top) GO and (bottom) EGNITE. The decrease of the oxygen signal indicates the reduction of the GO film. **i**, Conductivity of the GO and EGNITE films.

The preparation of EGNITE films is shown in Fig. 1a and further described in Methods. In brief, the micrometre-thick EGNITE film is obtained by vacuum filtration of an aqueous solution of graphene oxide (GO) flakes through a porous membrane, forming a free-standing GO film that is then transferred on top of the final substrate and hydrothermally reduced.

The structure of the EGNITE film consists of horizontally stacked flakes as revealed by scanning electron microscopy (SEM) (Fig. 1b). Following the hydrothermal reduction process, the stacking distance decreases from 8.1 ± 0.8 Å to 3.9 ± 0.6 Å, as assessed by X-ray diffraction (Fig. 1c). The stacking distance reduction is attributed to the removal of oxygen groups from the basal plane of the flakes^32^. The nanostructured cross section of EGNITE was further investigated by high-resolution transmission electron microscopy (HRTEM) (Fig. 1d). HRTEM data confirmed the stacked configuration of the flakes and the presence of nanometre-scale pores that form capillaries between flake planes, extending across the bulk of the material (Supplementary Fig. 1).

Understanding the topography and chemical composition of the outer surface is of particular importance due to its intended use in direct contact with biological tissue. A surface with a root mean square (r.m.s.) roughness of about 50 nm was determined by atomic force microscopy (AFM) measurements (Fig. 1e). Raman spectrograms reveal a higher defect content (*I*_D_/*I*_G_ ratio) in EGNITE compared with untreated GO (Fig. 1f).This can be explained by the effect of the hydrothermal reduction, which is assumed to pull out part of the basal plane, thus creating holes in the reduced GO flakes that we believe are at the origin of the highly enlarged electrochemical surface area of the material^33^.

The outer chemical composition of EGNITE was studied by X-ray photoelectron spectroscopy (XPS; Fig. 1g,h and Supplementary Fig. 2), which confirms the reduction process during the hydrothermal treatment, increasing the C/O ratio in the GO film from 2.4 to 3.8 (ref. ^34^). Electron energy-loss spectroscopy (EELS) was used to assess the chemical composition deep inside the material, revealing carbon and oxygen relative atomic contents of 85% and 15%, respectively (Supplementary Fig. 3). Moreover, the hydrothermal reduction process led to a decrease in the graphene-based film resistivity (Fig. 1i)^35^.

**Fig. 2:**
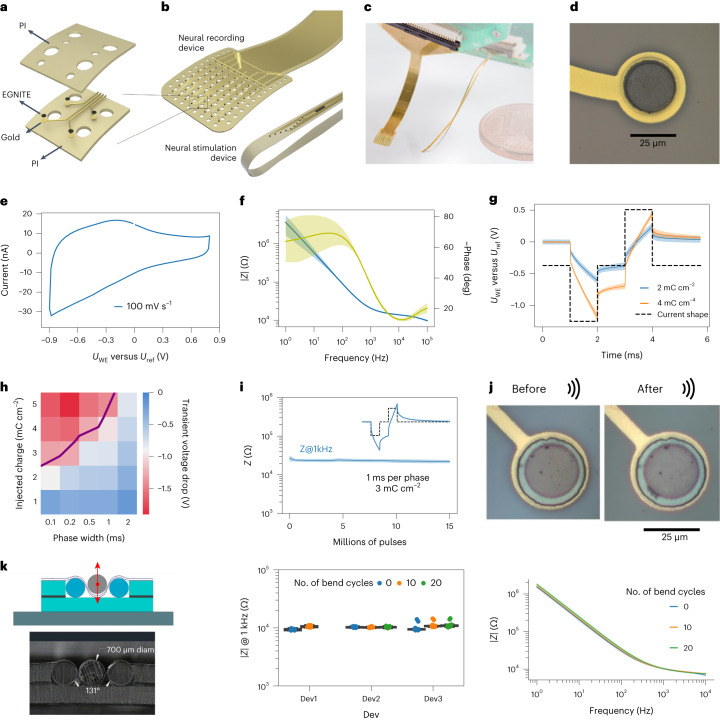
Microfabrication, electrochemical and structural assessment of microelectrodes based on EGNITE. **a**, Layers involved in the microfabrication of flexible arrays of microelectrodes of EGNITE. **b**, Designs of epicortical and intraneural arrays used in this work. **c**, Photograph of flexible implants based on EGNITE. **d**, Detailed micrograph of an EGNITE microelectrode of 25 µm diameter. **e**, Representative CV at 100 mV s^−1^ of an EGNITE microelectrode. **f**, EIS of EGNITE microelectrodes, showing the module (blue line) and phase (yellow line) of the impedance versus frequency. *n* = 18 electrodes. **g**, Voltage response to current-controlled biphasic pulses of 1 ms per phase (dashed lined) applied through EGNITE electrodes, corresponding to charge injection values of 2 and 4 mC cm^−^^2^. **h**, Map of the cathodic capacitive voltage excursion occurring at the interface between EGNITE microelectrodes and the electrolyte during the injection of current pulses at different levels of injected charge and pulse widths. **i**, Evolution of impedance at 1 kHz throughout continuous stimulation with 15 µA (3 mC cm^−^^2^) biphasic pulses of 1 ms per phase. **g**–**i**, *n* = 3 electrodes. **j**, Images of EGNITE electrodes fabricated in a PI device before and after 15 min of ultrasonication. **k**, Left: schematic and cross section of the bending set-up, indicating the pressure of a cylindrical bar of 700 µm diameter producing bending angles of 131°. Centre: impedance magnitude at 1 kHz for three different devices, each with 18 microelectrodes, measured before and after 10 and 20 bending cycles. Right: impedance spectrum for a device before and after 10 and 20 cycles of bending. Line represents the mean and shading the s.d. In **f**,**g**,**i**,**k**, data are mean (solid lines) ± s.d. (shaded area). In boxplots, the median, quartile box and minimum and maximum values (excluding outliers) are presented.

To exploit EGNITE for neural interfacing, we developed a wafer-scale fabrication process to integrate arrays of EGNITE microelectrodes into flexible devices (Fig. 2a and Methods). Polyimide (PI) is used as substrate and insulation layer^36,37^, and gold is used for the tracks. The procedure, described in Supplementary Fig. 4, results in high-yield flexible arrays of EGNITE electrodes of ~12 µm thickness. Figure 2b shows two designs, a 64-channel microelectrocorticography (µECoG) array organized in an 8 × 8 grid with a pitch of 300 µm and a transverse intrafascicular multichannel electrode (TIME) device with two linear arrays of nine microelectrodes each separated by 135 µm. The devices are flexible (Fig. 2c)^29,36^, and contain 25-µm-diameter EGNITE microelectrodes (Fig. 2d).

## In vitro electrode performance

The electrochemical performance of the 25-µm-diameter EGNITE microelectrodes was assessed in phosphate-buffered saline (PBS) solution (Methods). Cyclic voltammetry (CV) was used to assess the electrochemical window of the EGNITE material which was determined to be between −0.9 and +0.8 V (versus Ag/AgCl) (Fig. 2e). We also characterized the electrodes using electrochemical impedance spectroscopy (EIS) (Fig. 2f and Supplementary Fig. 7). The interfacial capacitance was estimated to be 13.9 mF cm^−^^2^, which corresponds to an ∼10^4^-fold increase with respect to the typical value of single-layer graphene (2 μF cm^−^^2^)^38,39^. At 1 kHz, the EGNITE microelectrodes exhibited an impedance of 25.2 ± 0.7 kΩ (*n* = 18).

The performance of EGNITE microelectrodes under current injection has also been studied. Figure 2g shows the electrode polarization that a 25-µm-diameter EGNITE microelectrode experiences upon the injection of cathodic-first rectangular, biphasic current pulses (1 ms per phase) at charge densities of 2.04 and 4.08 mC cm^−^^2^. We then determined the CIL of EGNITE electrodes under different pulse durations. Figure 2h shows a map of the microelectrode voltage polarization in response to biphasic current pulses of between 0.1 ms and 1 ms, for injected charge densities up to 5 mC cm^−^^2^.

The stability of the electrodes was investigated during continuous electrical stimulation. EGNITE microelectrodes were stimulated at a clinically relevant frequency (100 Hz) and observed to be stable after 15 million pulses as indicated by the rather constant impedance at 1 kHz (Fig. 2i), with no obvious structural changes observed (Supplementary Fig. 8). We also investigated the mechanical stability of EGNITE electrodes by sonication of the devices immersed in an ultrasound water bath^16^. After consecutive sonication for 15 min at 200 W and 300 W, the EGNITE electrodes remained attached to the device (Fig. 2j and Supplementary Fig. 9). No delamination or cracking of the electrodes was observed. To further evaluate device functionality under mechanical stress, a bending test around a rod of 0.7 mm diameter was performed. Over 98 ± 3% of the electrodes remained functional after ten bending cycles, and of those, all sustained ten additional bending cycles. Minor changes were observed in the impedance (Fig. 2k and Supplementary Fig. 10), indicating the mechanical stability of the EGNITE microelectrodes.

## In vivo brain signal recording

**Fig. 3:**
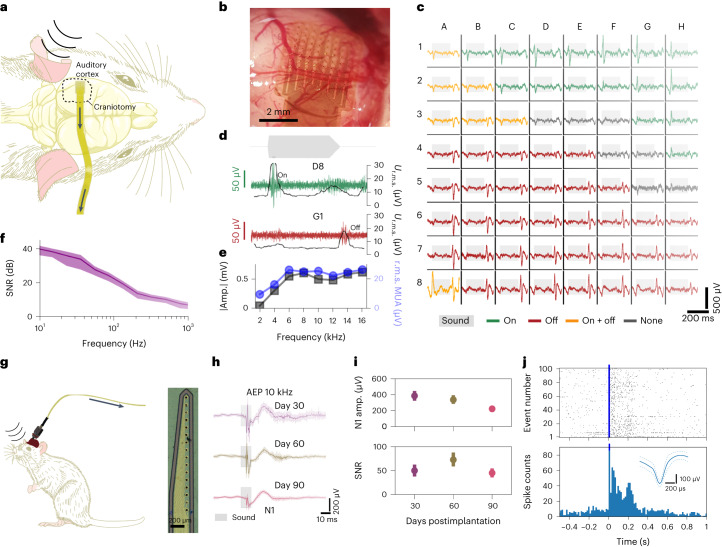
EGNITE-based μECoG arrays map with high spatiotemporal fidelity local field potentials and MUAs. **a**, Schematic diagram of the acute experiment using an EGNITE µECoG flexible array to record epicortical neural activity of a rat. Evoked activity was induced by pure tone stimuli. **b**, EGNITE µECoG array on the auditory cortex of a rat. **c**, Mapping of the evoked neural activity in response to 16 kHz stimuli, depicting a single event across all 64 EGNITE microelectrodes. Depending on the region, onset (green), offset (red), both (yellow) or no onset/offset (dark grey) responses are recorded. **d**, Response of regions G1 (onset, green) and D8 (offset, red) and HPF at 200 Hz to reveal high-frequency MUA activity (green and red), confirmed by a simultaneous increase in the r.m.s. value of the signal (grey). **e**, Maximum amplitude of the responses to sound stimuli at 2, 4, 6, 8, 10, 12, 14 and 16 kHz for the LFP HPF signal at 10 Hz (grey lines) and the averaged r.m.s. value of the signals above 200 Hz (r.m.s. MUA, blue lines), *n* = 15 stimuli. **f**, SNR (dB) calculated from the ratio of the in vivo and the post-mortem signals. Data are mean (solid line) ± s.d. (shaded area). **g**, Left: schematic diagram of intracortical flexible array configuration to record neural activity about 1.7 mm deep into the prefrontal cortex with EGNITE electrodes. Right: photograph of the microelectrode array tip. **h**, Averaged AEPs recorded 30, 60 and 90 days postimplantation. Data are mean (solid line) ± s.d. (shaded areas), *n* = 30 events. **i**, Top: peak N1 voltage change throughout 90 day intracortical device implantation. Bottom: SNR of the events. Data are mean ± s.d., *n* = 30 events. Data (single animal) recorded from same electrode are shown at days 30, 60 and 90. **j**, At day 30 postimplantation, there is robust spiking activity after the tone. Raster plot (top), peristimulus time histogram (bottom) and mean waveform (inset) of an individual neuron for 100 tone stimuli.

The suitability of the EGNITE microelectrodes for measuring neural signals was assessed by using flexible μECoG devices (Fig. 2b,c) to monitor cortical activity in anaesthetized rats (Fig. 3a). The probe was epicortically positioned over the left auditory cortex (primary auditory cortex and anterior auditory field regions) (Fig. 3b). Figure 3c (and Supplementary Fig. 11) shows the 10 Hz high-pass-filtered (HPF) signal from each of the 64 EGNITE electrodes in the array over a 350 ms window in which a pure tone was presented. Negative and positive evoked local field potentials (eLFPs) can be observed after the onset and offset of the sound stimulus^40^.

Figure 3d shows the same signals from two exemplary regions HPF at 200 Hz, together with the r.m.s. value. The high-frequency MUA and the corresponding increase of the r.m.s. amplitude due to MUA correlate well with the eLFP, indicating a synchronous behaviour of the neurons to the stimulus. Figure 3e shows the average response to sound stimuli at different frequencies for the LFP HPF signal at 10 Hz (grey lines) and the averaged r.m.s. value of the signals above 200 Hz (r.m.s. MUA, blue lines). Stimuli at 2 or 4 kHz elicited smaller responses, both in LFP and MUA, than stimuli above 6 kHz, as expected from the sound sensitivity of rats^41–43^.

The intrinsic r.m.s. noise of the electrode (calculated from post-mortem recordings) was 2.5 μV, very close to the limit of the electronic set-up^8,44^. Fig. 3f shows the signal-to-noise ratio (SNR) of the in vivo to post-mortem power spectral density (PSD) signals. The SNR reaches 40 dB at 10 Hz and 5 dB at 1 kHz (Fig. 3f), demonstrating high-fidelity recordings at both low and high frequencies^45^, outperforming the SNR obtained with a commercial platinum µECoG array (Supplementary Fig. 12).

Additionally, a proof-of-concept chronic recording experiment (Methods) was performed using an EGNITE intracortical device implanted in the prefrontal cortex in a mouse for over 90 days (Fig. 3g–j). After the first month, auditory evoked potentials (AEPs) were recorded longitudinally over the 3 months of implantation while the animal was freely moving. The observed AEP peak at 40 ms post stimulus was detected with mean SNR above 30 dB at all timepoints (Fig. 3h,i). Single-unit action potentials could be detected by the chronically implanted EGNITE electrodes at 1 month postimplantation (Fig. 3j).

## In vivo stimulation of nerve fibres

**Fig. 4:**
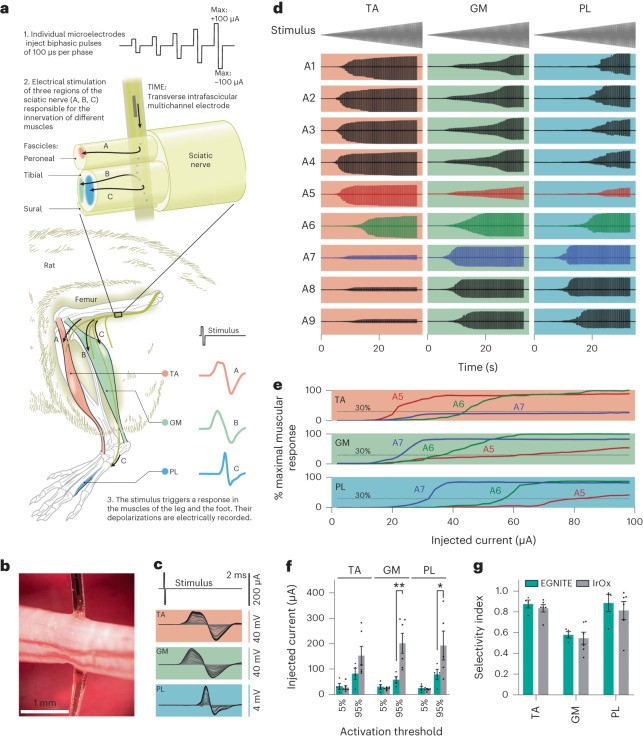
In vivo neural stimulation of peripheral nerve. **a**, Schematic diagram of the acute stimulation experiments. A TIME array is implanted in the sciatic nerve of the rat crossing the peroneal and the tibial fascicles. The axons innervating the TA muscle are in the peroneal fascicle, whereas the axons innervating the GM and PL muscles are located in the tibial fascicle. Biphasic pulses of 100 µs per phase and increasing intensity were injected independently at the nine microelectrodes (diameter, 25 µm) present in the device. The local electrical stimulation can depolarize the nerve fibres in its vicinity and trigger the electrical activity of the muscles, which is recorded with needle electrodes. **b**, Optical micrograph of the implanted TIME device in the sciatic nerve. **c**, CMAPs recorded from TA, GM and PL muscles in response to increasing levels of injected current pulses applied to one of the electrodes. **d**, Recorded CMAPs in TA, GM and PL muscles in response to trains of biphasic current pulses of increasing amplitude applied to nine microelectrodes of the array (A1–A9). **e**, Normalized CMAP of TA, GM and PL muscles in response to pulses injected to microelectrodes A5–A7 from the implanted TIME. **f**, Comparative plot of the injected current needed to elicit 5% and 95% of the maximum CMAP using microelectrodes of EGNITE (cyan, *n* = 4) and of iridium oxide (grey, *n* = 6)^48^. *P = 0.039, **P = 0.0032 (two-way ANOVA followed by Bonferroni post hoc test for differences between groups). **g**, Comparative plot of the selectivity index at the minimal functionally relevant muscular stimulation for the same data as in **f**^48^. In **f**,**g**, data are mean ± s.e.m.

The stimulation capability of the EGNITE microelectrodes was investigated using an array of transverse intrafascicular multichannel electrode (TIME)^46^ devices implanted in the sciatic nerve of anaesthetized rats (Fig. 4a). The device consisted of two linear arrays (A and B) of nine electrodes (diameter, 25 μm) along a 1.2 mm stripe. Each linear array faced opposite sides of the stripe. Once implanted (Fig. 4b), the device crossed the peroneal fascicle (responsible for the innervation of the tibialis anterior (TA) muscle) and the tibial fascicle (responsible for the innervation of both the gastrocnemius (GM) and plantar interosseous (PL) muscles). Each electrode in the EGNITE array was individually stimulated and the elicited compound muscle action potentials (CMAPs) of TA, GM and PL muscles were simultaneously recorded by monopolar needles in the muscles^46^ (Fig. 4c).

To stimulate the nerve, trains of 100 biphasic pulses (100 µs per phase) with increasing current amplitude (0 to 100 µA, in 1 µA steps) were used. Figure 4d shows the response of TA, GM and PL muscles to the current pulses applied through the microelectrodes A1–A9. Currents as low as 15–20 µA elicited CMAPs that increased in amplitude until a maximum activation was reached. The recruitment curves reflect the typical sigmoidal shape (Fig. 4e)^46,47^. The pattern of muscular activation changed depending on the stimulating electrode. The activity could be split in two clusters: one (A1–A5) in which the TA muscle was activated at lower stimulus intensity than the GM and the PL muscles, and another (A7–A9) in which the GM and PL muscles exhibited more activity than the TA muscle. This suggests that the first five microelectrodes were placed in the peroneal fascicle, whereas the last three were located within the tibial fascicle. From these responses, critical parameters for implants aiming at restoring mobility, such as the current thresholds and selectivity index of the evoked muscular activity, can be derived^3^.

Using 30% of muscle activation (the minimum stipulated to overcome gravity^46^) as a benchmark, the current stimulation at which this occurred for each muscle was determined for the TA, GM and PL muscles as marked in Fig. 4e. From these data, a selectivity index (SI_30%_) of >0.85 was calculated for the TA, 0.77 for the GM, while for the PL it was only 0.44. As an indicator, a maximal selectivity index of 1 indicates that one muscle can be solely activated without any activation in the other recorded muscles^3,9^.

A more detailed study of the activation threshold and selectivity was performed with TIME devices acutely implanted in rats. Figure 4f shows the mean values for the minimum current needed to achieve a 5% and a 95% of the maximal CMAP amplitude in the TA, GM and PL muscles. Less than 50 μA and 100 μA were required to reach 5% and 95% of maximal muscle activation, respectively. Compared to previous studies in which TIME devices with 80-µm-diameter electrodes of iridium oxide were used^48^, the 25-µm-diameter EGNITE electrodes elicited a response with current thresholds substantially lower (Fig. 4f).

Regarding the selectivity indices, high selectivity values close to 0.9 were obtained for TA and PL while selectivity for GM was lower (<0.6) (Fig. 4g)^48,49^. The higher selectivity obtained for the TA muscle can be explained by the separation of the fascicles by the perinerium as TA is innervated by axons in the peroneal fascicle while PL and GM are innervated by axons in the tibialis fascicle. Similarly, lower selectivity indices for GM (versus PL) can be explained due to the difficulties associated with obtaining intrafascicular selectivity, as both muscles are innervated by axons in the tibial fascicle, thus closer and without a perineurial barrier^49^.

## Biocompatibility of EGNITE microelectrodes

Chronic biocompatibility of the EGNITE-based devices was investigated in vivo both in the central and in the peripheral nervous system.

### Cortical biocompatibility

Chronic biocompatibility was studied with flexible epicortical devices implanted on adult rats for up to 12 weeks before histological and immunohistochemical evaluation of microglia phenotype morphology or extraction of cortical brain tissue for cytokine expression (by enzyme-linked immunosorbent assay (ELISA)). Control devices consisting of exposed gold electrodes and PI substrate, and gold passivation layers and PI-only devices were used to compare with the EGNITE devices at three time points postimplantation (2, 6 and 12 weeks; *n* = 3 per group). Moreover, cytokines released in the implanted and contralateral (no implantation) hemispheres were compared.

**Fig. 5:**
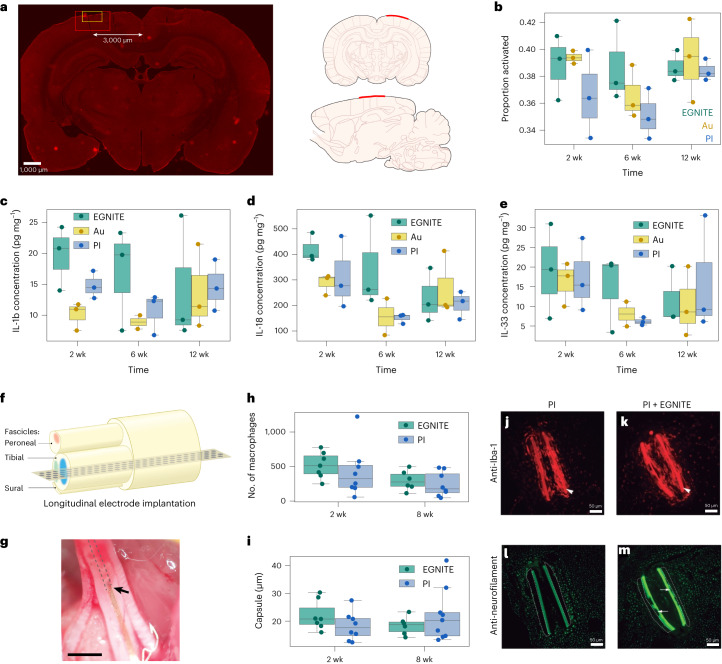
Biocompatibility of EGNITE technology in brain and sciatic nerve. Cortical tissue response. **a**, Left: representative brain section, postimplantation (left hemisphere) immunohistochemically stained for Iba-1. White arrow indicates the area of interest that was set at 3,000 µm from midline, based on average centre of device implantation. Red box: 8× magnification area used for microglial phenotype morphological analysis. Yellow box: 20× magnification area used to take representative images for the Iba-1 analysis in Supplementary Fig. 14. Right: EGNITE device implant location shown by the red line on coronal and sagittal rat brain sections. **b**, Iba-1 signal based on image processing of immunohistochemical sections. **c**–**e**, ELISA-based quantification of anti-inflammatory and proinflammatory cytokine levels (IL-1b (**c**), IL-18 (**d**), IL-33 (**e**)) normalized to total protein content (mg ml^−1^) of the tissue samples in direct contact with the EGNITE microelectrodes for 2, 6 and 12 weeks following their epicortical implantation. *n* = 3 for each device material. Peripheral nerve tissue response. **f**, Schematic of the intraneural biocompatibility experiment. A PI device with and without EGNITE is implanted in the tibial branch of the sciatic nerve of rats. **g**, Optical micrograph of the longitudinally implanted device in the rat sciatic nerve. Scale bar, 1 mm. The arrow indicates the insertion point, and the dashed lines indicate the placement of the intraneural device within the tibial fascicle. **h**, Number of inflammatory Iba-1-positive cells in the tibial nerve after 2 and 8 weeks of longitudinal implantation. **i**, Tissue capsule thickness formed around the implanted device. Boxplots, *n* = 7 for each device material. **j**–**m**, Representative images of transverse sections of a tibial nerve at 8 weeks after implantation of biocompatibility devices, made of PI alone (**j**,**l**) or PI with EGNITE (**k**,**m**), stained for inflammatory cells (antibody against Iba-1, **j**,**k**) and for axons (antibody against neurofilament 200, **l**,**m**). The arrowhead points to the transverse sections of the PI strips that were longitudinally inserted in the nerve. The arrows point to a site with EGNITE in the PI strip in **m**. The tissue capsule is delineated as dotted lines in **l** and **m**. Scale bar, 50 µm. The number of samples for the histological analyses was *n* = 6–7 per condition and time postimplantation. In boxplots, the median, quartile box and minimum and maximum values (excluding outliers) are presented.

Immunohistochemical analysis by Iba-1 (microglia marker) was performed to assess the level of microglial cell activation in the region directly in contact with the implanted devices (Fig. 5a and Supplementary Fig. 13). These results indicated no significant differences in the levels of microglia activation, regardless of electrode material or time point (Fig. 5b). Moreover, no histopathological sign of fibrotic tissue formation was observed in the area of the cortex in direct contact with the devices (Supplementary Fig. 14). No significant changes in the levels of inflammatory cytokines (including those involved in astrocytosis) from the cortical tissue samples at each time point (or between the different device types) were found (Fig. 5c–e and Supplementary Fig. 15). A minor increase in interleukin (IL)-1b, IL-6 and monocyte chemoattractant protein-1 (MCP-1) can be seen in the 2 and 6 week EGNITE-implanted groups compared to the groups with control devices (gold or PI). This difference is not seen at the 12 week time point (Fig. 5c and Supplementary Fig. 15). When compared to the contralateral hemisphere at each time point, no appreciable differences could be seen for any of the cytokines quantified, with the exception of the anti-inflammatory marker IL-18 (Fig. 5d) which was significantly increased at 2 weeks following implantation with EGNITE compared with the contralateral hemisphere. However, by 12 weeks this reaction also abated to background levels.

### Peripheral nerve biocompatibility

An intraneural device was designed in which the area of EGNITE in contact with the nerve was increased by a factor of 20, with the aim of maximizing the contact area to investigate immune responses. Intraneural implants, with EGNITE and control devices (PI-only devices), were longitudinally implanted in the tibial branch of the sciatic nerve of rats (Fig. 5f,g)^50^. Electrophysiological, pain and locomotion functional studies, and immunohistochemical labelling of the nerve, conducted at 2 and 8 weeks postimplantation and compared to the contralateral nerve or paw did not show any significant difference (Methods and Supplementary Fig. 16), indicating that there was no damage to myelinated motor nerve fibres by any of the implants, no damage of small nerve fibres or irritation induced by nerve compression or axonal injury and no functional nerve damage by the intraneural implants.

One of the main events during the foreign body reaction (FBR) is the infiltration by haematogenous macrophages into the implanted site, as part of the inflammatory phase^51^. Comparison between implants with and without EGNITE revealed no differences in the amount of macrophages in the nerve (Fig. 5h,j,k). The last phase of the FBR and one the main obstacles for long-term functionality of intraneural electrodes is the formation of a fibrous capsule around the implant. We analysed the fibrous capsule formation around the implant at different time points. Figure 5h shows that the capsule thickness formed around the PI strips was similar for implants with and without EGNITE at both 2 and 8 weeks, indicating that EGNITE does not induce damage to the nerve or further fibrotic scar formation. The time course of macrophage infiltration and capsule thickness showed a peak at 2 weeks and slight reduction at 8 weeks, as previously reported^50^. Immunohistochemical images (Fig. 5j–m) show numerous axons near the interneural implants (at around 20 µm) without and with EGNITE, indicating limited damage and remodelling after implant, consistent with previous works^50^.

## Conclusions

The EGNITE technology described here offers an attractive combination of properties that fulfil the requirements for next-generation neural interfaces. Its highly porous structure results in a high surface-to-volume ratio, resulting in a capacitance per geometric surface area of >10 mF cm^−^^2^, an ∼10^4^ increase compared with non-porous graphene electrodes. We have demonstrated that the EGNITE material can be miniaturized to the micrometre scale and integrated into microfabricated flexible arrays while preserving material properties. The developed microelectrode arrays exhibit high yield (>95%) and homogeneity and can deliver stimulation at high charge density (3–5 mC cm^−^^2^) over millions of pulses.

Surface and depth brain recordings demonstrate that, despite their reduced size (25 µm), the EGNITE microelectrodes exhibit low intrinsic noise levels, in the range of the instrumentation limits. EGNITE microelectrodes can record neural signals with high fidelity and high spatial resolution, exhibiting high SNR (>10 dB) for local field potential recordings. MUA and single-unit activity (SUA) was also recorded with high SNR, and a proof-of-concept chronic intracortical experiment showed LFP, MUA and SUA recordings over 1 month. Acute in vivo stimulation studies in the rat sciatic nerve were conducted to assess the stimulation capabilities of the technology. The small diameter and high density of EGNITE electrodes permitted focal stimulation with high selectivity and low charge stimulation thresholds (<100 µA) required for muscle activation.

Additionally, long-term biocompatibility and tissue functionality assessments conducted in the cortex and peripheral nerves suggest that the EGNITE technology is well tolerated, with minimal local or systemic tissue responses. This allows further development of the EGNITE microelectrode technology in applications that would require chronic implantation, including the determination of the degree of fibrotic capsule formation on chronic functional use of the devices.

The evaluation of chronic functionality and stability of EGNITE electrodes in a neuromodulation therapeutic setting^25^ will require further investigation. Considering graphene’s chemical stability and wide electrochemical potential window, we expect improvements over alternative polymeric and metal-based electrode materials. Future research could focus on further improvement of its electrochemical performance. Long-term stability aspects related to the microfabrication process should also be explored, such as eliminating the metal back-contacts to prevent long-term corrosion processes^52^.

The high SNR obtained with EGNITE microelectrodes allows for high-fidelity brain mapping, therefore potentially improving brain signal decoding^53^ and facilitating the discovery of new neural biomarkers^54^. Moreover, the low charge stimulation threshold observed with the intraneural microelectrodes can be particularly suited for providing sensory feedback in applications in which precise control of motor prostheses is needed^4^ or for high-resolution deep-brain stimulators or retinal neuroprostheses^14,15,55,56^. In addition, low charge stimulation thresholds can eventually lead to lower power consumption of chronically implanted stimulators, hence extending battery lifetimes and facilitating wireless powering.

The graphene-based technology presented here, integrated into microfabrication processes reproducibly and with the capacity to be upscaled, will be critical to achieve clinical translation and comply with the stringent regulatory requirements for invasive clinical applications. The combination of high-performance electrical stimulation and neural recording capabilities, coupled with chronic tissue tolerance, indicates that the thin-film EGNITE microelectrode technology can contribute to the next generation of bidirectional neural interfaces.

## Methods

### Material preparation and characterization

Aqueous GO solution was diluted in deionized water to obtain a 0.15 mg ml^−1^ solution and vacuum filtered through a nitrocellulose membrane with pores of 0.025 µm, forming a thin film of GO. The thin film was then transferred to the target substrate using wet transfer in deionized water and further thermal annealing at 100 °C for 2 min. The GO film–substrate stack was hydrothermally reduced at 134 °C in a standard autoclave for 3 h to form EGNITE. The base substrate for all characterization studies of EGNITE was a square (1 × 1 cm^2^) of Si/SiO_2_ (400 μm/1 μm).

#### XPS

XPS measurements were performed with a Phoibos 150 analyser (SPECS) in ultra-high-vacuum conditions (base pressure, 5 × 10^−^^10^ mbar) with a monochromatic Al Kα X-ray source (1,486.74 eV). Overview spectra were acquired with a pass energy of 50 eV and step size of 1 eV and high-resolution spectra were acquired with pass energy of 20 eV and step size of 0.05 eV. The overall resolution in those last conditions is 0.58 eV, as determined by measuring the full width at half maximum of the Ag 3*d*5/2 peak of sputtered silver. The XPS analysis shows a strong decrease after the hydrothermal treatment of the C–O peak (associated with epoxide groups), but a small contribution of C–OH, C=O and C(O)OH due to hydroxyls, carbonyls and carboxyls that remain after reduction. The deconvolution of the O1*s* peak confirms such behaviour. The main contribution to the C1*s* signal after the hydrothermal reduction, however, comes from *sp*^2^ hybridized C–C orbitals^34,57^.

#### X-ray diffraction

X-ray diffraction measurements (*θ*–2*θ* scan) were performed in a Materials Research Diffractometer (Malvern PANalytical). This diffractometer has a horizontal *ω*–2*θ* goniometer (320 mm radius) in a four-circle geometry and worked with a ceramic X-ray tube with Cu Kα anode (*λ* = 1.540598 Å). The detector used is a Pixcel which is a fast X-ray detector based on Medipix2 technology.

#### Raman spectroscopy

Raman spectroscopy measurements were performed using a Witec spectrograph equipped with a 488 nm laser excitation line. For the measurements, Raman spectra were acquired using a 50× objective and a 600 grooves per nm grating; laser power was kept below 1.5 mW to avoid sample heating.

#### TEM

A focused ion beam lamella was prepared with a Helios NanoLab DualBeam (LMA-INA) for the cross-section study of the EGNITE sample. Structural analyses were performed by means of TEM using a Tecnai F20 microscope operated at 200 kV, including HRTEM and high-angle annular dark-field STEM techniques. The STEM-EELS experiment was performed in a Tecnai F20 microscope working at 200 KeV, with 5 mm aperture, 30 mm camera length, a convergence angle of 12.7 mrad and a collection angle of 87.6 mrad. As we used 0.5 eV per pixel and 250 eV as the starting energy in the core-loss acquisition, we did not acquire the Si K-edge expected at 1,839 eV, the Pt M-edge at 2,122 eV and the Au M-edge at 2,206 eV. The relative C–O atomic composition has been obtained by focusing our attention in the reduced GO layer and assuming that the edges analysed (C and O in our case) sum to 100%. This assumption is valid in our case as evidenced in the Supplementary Information maps. The energy differential cross section was computed using the Hartree–Slater model and the background using a power-low model.

#### Electrical conductivity

Electrical conductivity measurements were performed using a Keithley 2400 sourcemeter in two-point configuration. The samples measured consisted of EGNITE films of 1 × 1 cm^2^ on top of a SiO_2_ substrate.

#### Data analysis

X-ray diffraction, Raman and XPS data were analysed using Python 3.7 packages (Numpy, Pandas, Scipy, Xrdtools, Lmfit, Rampy, Peakutils, Matplotlib). The distance between planes was calculated from the X-ray diffraction measurements according to Snell’s law. Once the data were moved into the spatial domain, the maximum of the peaks was fitted. The corresponding distance gave a mean value of the distance between planes. Deviations from those mean values were calculated from the full width at half maximum of the Lorentzian fittings of the peaks on the spatial domain. XPS and Raman spectroscopy measurements were analysed by fitting a convolution of peaks on expected locations for the corresponding features. The conductivity values of the GO and EGNITE were obtained by fitting the *I*–*V* curves measured in the electrical conductivity measurements to Ohm’s law. Data are *n* = 1 for each measurement.

### Flexible array fabrication

The fabrication of the devices is shown in Supplementary Fig. 4. Devices were fabricated on 4 inch Si/SiO_2_ (400 μm/1 μm) wafers. First, a 10-µm-thick layer of PI (PI-2611, HD MicroSystems) was spin coated on the wafer and baked in an atmosphere rich in nitrogen at 350 °C for 30 min. Metallic traces were patterned using optical lithography of the image reversal photoresist (AZ5214, Microchemicals). Electron-beam evaporation was used to deposit 20 nm of titanium and 200 of gold and lift-off was performed. We used an EGNITE film of around 1 μm thickness as a trade-off between electrochemical performance and array flexibility. After transferring the GO film, aluminium was e-beam evaporated and areas on top of the future microelectrodes were defined by using a negative photoresist (nLOF 2070, Microchemicals) and lift off. Next, the GO film was etched everywhere apart from the future microelectrodes using an oxygen reactive ion etching (RIE) for 5 min at 500 W and the protecting aluminium columns were etched with a diluted solution of phosphoric and nitric acids. Then, a 3-µm-thick layer of PI-2611 was deposited onto the wafer and baked as previously described. PI-2611 openings on the microelectrode were then defined using a positive thick photoresist (AZ9260, Microchemicals) that acted as a mask for a subsequent oxygen RIE. Later, the devices were patterned on the PI layer, again using AZ9260 photoresist and RIE. The photoresist layer was then removed in acetone and the wafer cleaned in isopropyl alcohol and dried out. Finally, the devices were peeled off from the wafer and were ready to be placed in sterilization pouches to be hydrothermally treated at 134 °C in a standard autoclave for 3 h.

### Microelectrode electrochemical characterization

Electrochemical characterization of the microelectrodes was performed with a Metrohm Autolab PGSTAT128N potentiostat in 1× PBS (Sigma-Aldrich, P4417) containing 10 mM phosphate buffer, 137 mM NaCl and 2.7 mM KCl at pH 7.4 and using a three-electrode configuration. An Ag/AgCl electrode (FlexRef, WPI) was used as reference and a platinum wire (Alfa Aesar, 45093) was used as counter-electrode.

Prior to performance evaluation, electrodes were pulsed with 10,000 charge-balanced pulses (1 ms, 15 µA). Exposure of electrodes to continuous pulsing protocols proceeded by 100 cyclic voltammetry cycles (−0.9 to +0.8 V) at 50 mV s^−1^, 20 repetitions of 5,000 pulses (1 ms) and redetermination of the open circuit potential.

#### Data analysis

Electrochemical characterization data were analysed using Python 3.7 packages (Numpy, Pandas, Scipy, Pyeis, Lmfit, Matplotlib). Impedance spectroscopy data were fitted to an equivalent electric model consisting of a resistance (*R*) in series with a constant phase element (CPE). From there, the CPE value was approximated to a capacitance and divided by the microelectrode geometric area to obtain an equivalent value for the interfacial capacitance of EGNITE. Microelectrode charge storage capacitance (CSC) was calculated from cyclic voltammetry measurements by integrating the cathodic and anodic regimes of the measured current and normalizing by the scan rate. The cathodic and anodic charge storage capacitance (cCSC and aCSC) at 100 mV scan rate of EGNITE are 45.9 ± 2.4 and 34.6 ± 2.8 mC cm^−2^, respectively (*n* = 3). As reported for other materials^58^, the obtained CSCs depend on the scan rate (Supplementary Fig. 5). To assess the presence of oxygen reduction reactions, we measured the CV waveform under nitrogen-purged electrolyte^59^ and did not observe substantial differences in waveform (Supplementary Fig. 6). However, our results do not fully address the impact of oxygen reduction reactions in the charge injection capacity of EGNITE and additional work needs to be done to properly investigate this. Microelectrode charge injection capacity (CIC) was established by determining the current pulse amplitude that elicited a voltage difference (after removing the ohmic drop) that matched the electrode electrochemical water window (−0.9 V for cathodic and +0.8 V for anodic versus Ag/AgCl) (Supplementary Fig. 17)^60^.

#### Statistical analysis

Data are mean ± s.d., *n* = 18 for EIS and *n* = 3 for chronopotentiometries. Data of the map of cathodic capacitive voltage excursion are the mean of the cathodic capacitive voltage excursions for one event for each pulse shape of *n* = 3 electrodes.

### Mechanical stability evaluation

#### Ultrasound sonication

EGNITE electrode arrays were placed inside a beaker filled with water in an ultrasound water bath (Elmasonic P 180H). Sonication was applied at 37 kHz for 15 min at 200 W, and followed by an additional 15 min of sonication at 37 kHz with the power elevated to 300 W. Images of electrodes were acquired before and after the sonication steps.

#### Bending test

The bending set-up (Fig. 2k) consisted of three cylindrical rods; the middle one (diameter, 700 µm) was lowered down, producing bending angles of 131°. Three flexible microelectrode arrays were used for the bending test. Each array contained 18 microelectrodes of 50 µm diameter. Two arrays were measured after 10 and 20 cycles while one device was measured only for 10 cycles as it was damaged during handling after measuring. The bending test cycle consisted of a 10-s-long load application plus 10 s with no load. Devices were electrochemically characterized (EIS and CV) before and after 10 and 20 bending cycles.

### Epicortical neural recording

#### Epicortical implantation

All experimental procedures were performed in accordance with the recommendations of the European Community Council and French legislation for care and use of laboratory animals. The protocols were approved by the Grenoble ethical committee (ComEth) and authorized by the French ministry (number 04815.02). Sprague–Dawley rats (male, 4 months old, weighing ∼600 g) were anaesthetized intramuscularly with ketamine (50 mg per kg (body weight)) and xylazine (10 mg per kg (body weight)), and then fixed to a stereotaxic holder. Removing the temporal skull exposed the auditory cortex. Dura mater was preserved to avoid damaging the cortical tissue. A hole was drilled at the vertex to insert the reference electrode, and a second hole, 7 mm toward the front from the first one, was drilled to insert the ground electrode. The electrodes were 0.5-mm-thick pins used for integrated circuit sockets. They were placed to make electrical contact with the dura mater and fixed to the skull with dental cement. We then mounted the surface microelectrode ribbon on the auditory cortex as shown in Fig. 3b. The vein patterns identify the auditory cortex, in area 41 of Krieg’s rat brain map. Cortical signals were simultaneously amplified with a gain of 1,000 and digitized at a sampling rate of 33 kHz. A speaker 20 cm in front of a rat’s ear, contralateral to the exposed cortex, delivered acoustic stimuli. The stimuli delivered were monitored by a 0.25 inch microphone (Brüel & Kjaer, 4939) placed near the ear and presented in sound pressure level (dB SPL re 20 μPa). We examine the vertex-positive (negative-up) middle-latency responses evoked by alternating clicks at 80 dB SPL, and tone burst stimuli at 70 dB SPL with frequencies ranging from 5 to 40 kHz, a rise and fall time of 5 ms and a duration of 200 ms.

#### Data analysis

Electrophysiological data were analysed using Python 3.7 packages (Numpy, Pandas, Scipy, Neo, Elephant, Sklearn Matplotlib) and the custom library PhyREC (https://github.com/aguimera/PhyREC). r.m.s. values were calculated with a sliding window of 20 ms at frequencies above 200 Hz. Spectrograms were calculated for a range between 70 Hz and 1.1 kHz. PSD was calculated over 60 s of continuous recordings. For a given electrode array, two PSDs were calculated: in vivo (IV) and post-mortem (PM). The SNR is expressed in dB (20 × ln(r.m.s.(IV)/r.m.s.(PM))) and interpolated for 20 points logarithmically spaced between 10 Hz and 1 kHz.

#### Statistical analysis

Epicortical neural data presented in Fig. 3 are taken from individual measurements on a single animal. In Fig. 3c, data from 64 electrodes are presented. In Fig. 3d, data from two selected electrodes are presented. In Fig. 3f, the PSD and SNR are calculated from 64 EGNITE electrodes and are shown as mean ± s.d. In Supplementary Fig. 12c,d median data are presented for 192 EGNITE electrodes from *n* = 3 experiments and 60 platinum electrodes from *n* = 1 experiment.

### Intracortical neural recording

#### Intracortical implantation

Animals were anaesthetized with a mixture of ketamine/xylazine (75:1, 0.35 ml/28 g i.p.) and this state was maintained with an inhalation mask providing 1.5% isoflurane. Several microscrews were placed into the skull to stabilize the implant, and the one on top of the cerebellum was used as a general ground. The probe was implanted in the prefrontal cortex (coordinates: AP, 1.5 mm; ML, ±0.5 mm; DV, −1.7 mm from bregma). The implantation was performed by coating the probe with maltose (see protocol below) to provide temporary probe stiffness and facilitate probe insertion. The probe was sealed with dental cement. TDT-ZifClip connectors were used to connect the probe to the electrophysiological system via a miniaturized cable. After the surgery, the mouse underwent a recovery period of 1 week receiving analgesia (buprenorphine) and anti-inflammatory (meloxicam) treatments. Neural activity was recorded with the multichannel Open Ephys system at a sampling rate of 30 kHz with an Intan RHD2132 amplifier. The auditory task experiments were conducted in a soundproofed box, with two speakers inside using protocols based on previously described work^61^. The sound stimulus consisted of a 15-ms-long white noise click, repeated 100 times (cycles), each separated by 5 s (interstimulus interval). During the task, the animal was able to move freely.

#### Maltose stiffener protocol

An aqueous solution of maltose is heated up to the glass transition point (*T*_g_), between 130 and 160 °C, using a hot plate or a microwave. Once the maltose is viscous, the backside of the probe is brought into contact only with the maltose. As the maltose cools down, it rigidifies and stiffens the probe.

#### Data analysis

Neural signals from each electrode were filtered offline to extract SUA and LFPs. SUA was estimated by filtering the signal between 450 and 6,000 Hz and the spikes from individual neurons were sorted using principal-component analysis with Offline Sorter v.4 (Plexon). To obtain LFPs, signals were downsampled to 1 kHz, detrended and notch-filtered to remove noise line artefacts (50 Hz and its harmonics) with custom-written scripts in Python. AEP SNR was calculated as the ratio of the peak N1 amplitude and the s.d. of a 20 ms period prior to the stimulus.

#### Statistical analysis

Data shown in Fig. 3h,i are mean ± s.d., *n* = 30 as the number of averaged trials. Data recorded from the same electrode are shown at days 30, 60 and 90. Data from a single animal are presented.

### Chronic epicortical biocompatibility

#### Surgical implantation of devices

A total of 27 adult, male, Sprague–Dawley rats were used for this study (Charles River). Animals were housed at an ambient temperature of 21 ± 2 °C and a humidity of 40–50%, on a 12 h light/12 h dark cycle. Rats were housed in groups and given free access to diet and water throughout the experimental period. Experimental procedures were carried out in accordance with the Animal Welfare Act (1998), under the approval of the UK Home Office and the local animal welfare ethical review body (AWERB). Animals were anaesthetized with isoflurane (2–3%) for the duration of surgery, and the depth of anaesthesia was monitored by the toe pinch reflex test. Animals were placed in a stereotaxic frame (Kopf, 900LS), located above a thermal blanket to maintain body temperature. A craniotomy hole (∼5 mm ×4 mm) was made 1 mm away from the midline using a dental drill with a 0.9 mm burr drill bit, the dura was removed and the epicortical device placed on the cortical surface of the brain. The craniotomy hole was sealed with Kwik-sil, followed by dental cement to secure, and the skin sutured closed. Subcutaneous injections of saline (1 ml per kg (body weight)) and buprenorphine (0.03 mg per kg (body weight)) were given to replace lost fluids and reduce postoperative pain, and anaesthesia was withdrawn.

#### Tissue collection and processing

Animals were terminated at 2, 6 or 12 weeks postimplantation by an appropriate method for the type of analysis to be performed.

#### Histology and immunohistochemistry

At 2, 6 or 12 weeks postimplantation rats were terminated via cardiac perfusion with heparinized (10 U ml^−1^, Sigma-Aldrich) PBS, followed by 4% paraformaldehyde (PFA, Sigma-Aldrich) in PBS. Brains were postfixed in 4% PFA for 24 h, then transferred to 30% sucrose in PBS for at least 48 h before freezing in isopentane. The brains were then stored at −80 °C until cryosectioned at 25 µm. The tissue was then stained for ionized calcium binding adaptor molecule 1 (Iba-1) to determine the level of microglial activation. Briefly, tissue sections were blocked with 5% goat serum in PBS with 0.1% Triton-X for 1 h before overnight incubation at 4 °C with the primary antibody anti-Iba-1 (1:1,000, 019-19741; Wako). Sections were then stained with secondary antibody, anti-rabbit Alexa Fluor 594 (1:400, A-11012; Thermo Fisher) for 1 h at room temperature. Slides were mounted with coverslips using Prolong Gold anti-fade mounting media with 4,6-diamidino-2-phenylindole (Thermo Fisher). The probe covered an area of 3 × 3.7 mm^2^ on the cortical surface of the brain; tissue sections selected for staining covered 3.2 mm in length of this region. Slides were imaged using a 3DHistech Pannoramic-250 microscope slide scanner at 20× and images were analysed using CaseViewer v.2.4 (3DHistech). To assess for microglia activation, a 3.2 mm area was covered, with one image analysed every 100 µm. Images were taken at 8.5× magnification which detailed a section of the epicortical probe site, 3 mm from the midline of the brain, encompassing the area directly under the probe site.

#### Image processing

The microscopy data were image-processed using an algorithm for microglia phenotype characterization (Supplementary Fig. 13). Microglial activation was analysed using a custom CellProfiler* (Broad Institute, v.3.1.9 from https://cellprofiler.org/) pipeline. First, the EnhanceOrSuppressFeatures module was used to enhance filamentous structures like neurites by applying the tubeness enhancement method. From the enhanced images, cells were segmented using the IdentifyPrimaryObjects module. Preliminary measurements of the cells suggested that the appropriate object diameter range was 3–40 pixels. Objects outside this diameter range or touching the edge of the image were discarded. The cells were segmented using a two-class Otsu adaptive thresholding strategy with an adaptive window size of 50 pixels. The objects identified by the IdentifyPrimaryObjects module were input to the MeasureObjectSizeShape module to calculate the necessary properties for cell classification. In the ClassifyObjects module, the category on which to base classifications was specified to be AreaShape, and Extent was selected as the corresponding measurement. The cells were classified as *‘*activated’ or ‘non-activated’ based on their Extent property, which is the ratio of the area occupied by the cell to the area occupied by its bounding box. This classification approach was rationalized by the fact that activated microglia have large cell bodies and no processes, and thus occupy a far larger proportion of their bounding boxes than their non-activated counterparts. Finally, the CalculateMath and ExportToSpreadsheet modules were used to calculate and output the desired statistics.

#### Statistical analysis

Data sets are *n* = 3 for each device type (PI-only implant (PI); PI with exposed microfabricated gold (gold); and PI with microfabricated gold and EGNITE (EGNITE) at all time points) with the exception of 6 week gold which is *n* = 2 for ELISA data. Contralateral hemispheres were combined at each time point to give *n* = 9 at 2 and 12 weeks postimplantation and *n* = 8 at 6 weeks postimplantation. Analysis of the data was done using GraphPad Prism v.8 software. Statistical analysis was completed using a two-way analysis of variance (ANOVA) with Tukey’s multiple-comparisons test where appropriate; *P* < 0.05 was deemed to be significant.

#### ELISA

Following the implantation period, animals were terminated by cervical dislocation. Brain tissue was extracted from both the right and left hemisphere of the brain, snap frozen in liquid nitrogen and stored at −80 °C until further use. Tissue was lysed using NP-40 lysis buffer (150 mM NaCl, 50 mM Tris-Cl, 1% Nonidet P40 substitute, Fluka, pH adjusted to 7.4) containing protease and phosphatase inhibitor (Halt Protease and Phosphatase Inhibitor Cocktail, Thermo Fisher), followed by mechanical disruption of the tissue (TissueLyser LT, Qiagen). Samples were then centrifuged for 10 min at 5,000 r.p.m., and the supernatant stored at 4 °C until further use. The LEGENDplex Rat Inflammation Panel (catalogue number 740401, BioLegend), a bead-based multiplex ELISA kit, was run to quantify the following cytokines; IL-1α, IL-1β, IL-6, IL-10, IL-12p70, IL-17A, IL-18, IL-33, CXCL1 (KC), CCL2 (MCP-1), granulocyte–macrophage colony-stimulating factor, interferon-γ and tumour necrosis factor. The kit was run according to the manufacturer’s instructions, with protein loaded at a fixed volume of 15 µl. Following incubation with supernatant the beads were run on a BD FACSVerse flow cytometer, and the data analysed using LEGENDplex data analysis software.

### Neural stimulation

#### Intrafascicular implantation

All animal experiments were approved by the Ethical Committee of the Universitat Autònoma de Barcelona in accordance with the European Communities Council Directive 2010/63/EU. Animals were housed at 22 ± 2 °C under a 12 h light/12 h dark cycle with food and water freely available. The sciatic nerve of anaesthetized female Sprague–Dawley rats (250–300 g, ∼18 weeks old) was surgically exposed and the TIME electrodes were implanted transversally across the sciatic nerve with the help of a straight needle attached to a 10-0 loop thread^46^. The process was monitored under a dissection microscope to ensure the correct position of the active sites inside the nerve fascicles (Fig. 4b). During the experiments, the animal body temperature was maintained with a heating pad.

Nerve stimulation was performed by applying trains of biphasic current pulses of a fixed duration of 100 µs per phase and increasing amplitude from 0 to 150 µA in 1 or 3 µA steps at 3 Hz for 33 s (Stimulator DS4, Digitimer) through the different EGNITE microelectrodes. Simultaneously, the CMAPs were recorded from GM, TA and PL muscles using small needle electrodes (13 mm long, 0.4 mm diameter, stainless steel needle electrodes A-03-14BEP, Bionic) placed in each muscle^62^. The active electrode was placed on the muscle belly and the reference at the level of the tendon. Electromyography recordings were amplified (×100 for GM and TA, ×1,000 for PL; P511AC amplifiers, Grass), band-pass filtered (3 Hz to 3 kHz) and digitized with a PowerLab recording system (PowerLab16SP, ADInstruments) at 20 kHz.

#### Data analysis

The amplitude of each CMAP was measured from baseline to the maximum negative peak. The voltage peak measurements were normalized to the maximum CMAP amplitude obtained for each muscle in the experiment. A selectivity index (SI) was calculated for each active site as the ratio between the normalized CMAP amplitude for one muscle, CMAP_*i*_, and the sum of the normalized CMAP amplitudes in the three muscles, following the formula SI_*i*_ = *n*CMAP_*i*_/∑*n*CMAP_*j*_, at the minimum stimulation current amplitude that elicited a minimal functionally relevant muscular response (defined as at least 5% CMAP amplitude for one of the muscles with the respect to the maximum CMAP amplitude of that muscle that had been previously determined). Then, the active sites with highest SI for each of the three muscles were selected as the SIs for each muscle in a given experiment.

### Chronic intraneural biocompatibility

Following a previously reported procedure^50,63^, the sciatic nerve of anaesthetized Sprague–Dawley female rats (250-300 g, ∼18 weeks old) was exposed and the devices for in vivo biocompatibility with and without EGNITE were longitudinally implanted in the tibial branch of the sciatic nerve (*n* = 6–8 per group). Briefly, the nerve is pierced at the trifurcation with a straight needle attached to a 10-0 loop thread (STC-6, Ethicon); the thread pulls the arrow-shaped tip of the bent electrode strip. The tip is cut to take away the thread, and the tips of each arm are slightly bent to avoid withdrawal of the device. A longitudinal implant was chosen because it allows a better study of the foreign body response inside the nerve^50^.

#### Nerve and animal functional assessment

Animals were evaluated during follow-up postimplantation by means of nerve conduction, algesimetry and walking track locomotion tests^62^. For conduction tests, the sciatic nerve of the implanted and contralateral paws was stimulated by needle electrodes at the sciatic notch and the CMAP of the PL muscle was recorded as above. The latency and the amplitude of the CMAP were measured. For the algesimetry test, rats were placed on a wire net platform and a mechanical non-noxious stimulus was applied with a metal tip connected to an electronic Von Frey algesimeter (Bioseb). The nociceptive threshold (force in grammes at which the animals withdrew the paw) of implanted versus contralateral paws was measured. For the walking track test, the plantar surface of the hindpaws was painted with black ink and each rat was left to walk along a corridor. The footprints were collected, and the sciatic functional index calculated^62^.

#### Histology

After 2 or 8 weeks, animals were perfused with PFA (4%), and the sciatic nerves were harvested, postfixed, cryopreserved and processed for histological analysis. For the evaluation of the FBR, sciatic nerves were cut in 15-μm-thick transverse sections with a cryostat (Leica CM190). Samples were stained with primary antibodies for myelinated axons (anti-RT97 to label Neurofilament 200K, 1:200; Developmental Studies Hybridoma Bank) and macrophages (anti-Iba-1, 1:500; Wako). Then, sections were incubated for 1 h at room temperature with secondary antibodies donkey anti-mouse Alexa Fluor 488 and donkey anti-rabbit Alexa Fluor 555 (1:200, Invitrogen). Representative sections from the central part of the implant in the tibial nerve were selected, images taken with an epifluorescence microscope (Eclipse Ni, Nikon) attached to a digital camera (DS-Ri2, Nikon) and image analysis performed with ImageJ software (National Institutes of Health). The amount of Iba-1-positive cells in the whole area of the tibial nerve was quantified and the thickness of the tissue capsule was measured as the mean distance of each side of the implant to the closest axons.

#### Statistical analysis

For statistical analysis of data, we used one- or two-way ANOVA followed by Bonferroni post hoc test for differences between groups or times. GraphPad Prism software was used for graphical representation and analysis. Statistical significance was considered when *P* < 0.05.

### Reporting summary

Further information on research design is available in the Nature Portfolio Reporting Summary linked to this article.

## Online content

Any methods, additional references, Nature Portfolio reporting summaries, source data, extended data, supplementary information, acknowledgements, peer review information; details of author contributions and competing interests; and statements of data and code availability are available at 10.1038/s41565-023-01570-5.

### Supplementary information

Supplementary Information Supplementary Figs. 1–17.

Reporting Summary

## Data Availability

All relevant data obtained to evaluate the main findings of the paper are openly available in Zenodo at 10.5281/zenodo.10208681. All other raw data are available from the corresponding author upon reasonable request.

## Code availability

Custom code developed for neurophysiological analysis is available at https://github.com/aguimera/PhyREC. Custom scripts can be found in Zenodo at 10.5281/zenodo.10208681.
