# Supplementary material for: Nanoporous graphene-based thin-film microelectrodes for in vivo high-resolution neural recording and stimulation
Source: Nat Nanotechnol. 2024 Jan 11;19(4):514–23. doi: 10.1038/s41565-023-01570-5 (PMC11026161; doi:10.1038/s41565-023-01570-5)
Supplement: Supplementary file 1 — Supplementary Figs. 1–17. [file 41565_2023_1570_MOESM1_ESM.pdf]

nature nanotechnology

Article

https://doi.org/10.1038/s41565-023-01570-5

Nanoporous graphene-based thin-film microelectrodes for in vivo high-resolution neural recording and stimulation

In the format provided by the authors and unedited

Supplementary Information

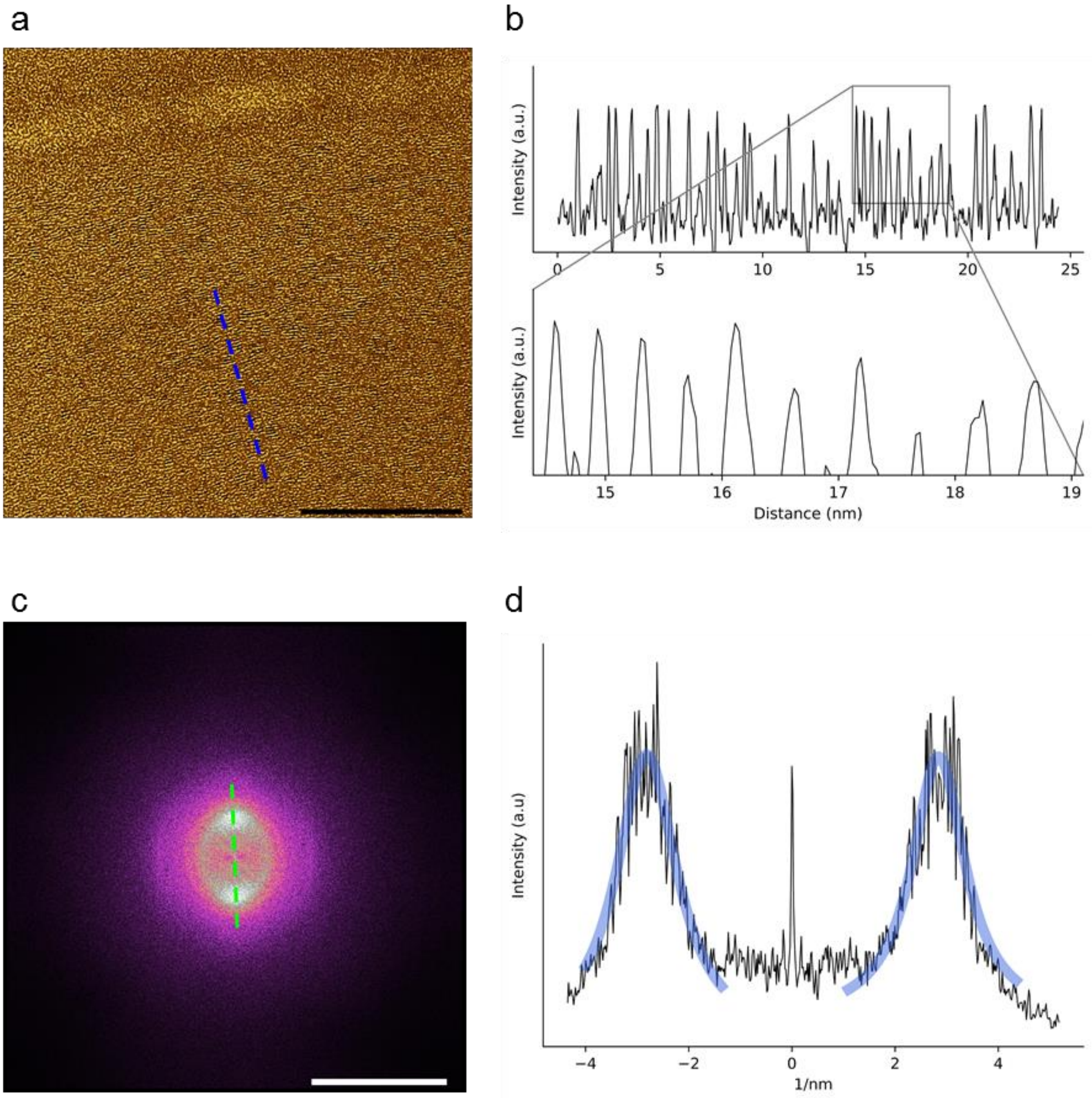

Figure SI 1. Transmission Electron Microscopy.

a. False color cross-sectional view of EGNITE taken by HRTEM along a cross section lamella of the material. Scale bar = 20 nm. **b.** Profile along the blue line in a. The intensity of the image oscillates periodically every 4 Å. **c.** Power spectrum of a. Scale bar = 1/10 nm. **d.** Profile along the central region, in the vertical direction from c. Two symmetric peaks appear at 1/2.7 nm indicating a preferential direction in the stacking of the flakes at 3.7 Å. Lorentzian functions can be fitted to the peaks, which suggests a distribution of the orientation of the flakes around the preferred direction.

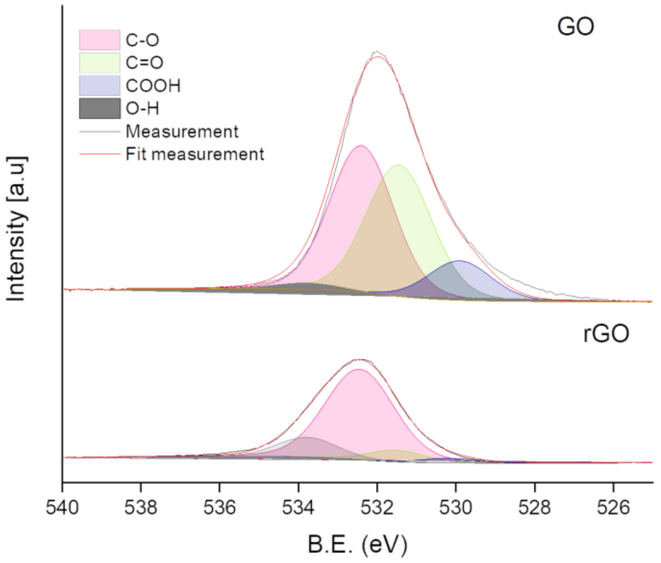

Figure SI 2. O1s peak deconvolution

High resolution measurement in the O1s peak by X-ray photoelectron spectroscopy (XPS) of GO and EGNITE films. The O1s peak was deconvoluted onto four peaks: CO(OH) at 530.5 eV, C=O at 531.6 eV, C-O at 532.4 eV and H-O 533.8 eV. The evolution of such peaks after the hydrothermal reduction confirms the decrease of the C=O bondings and the increase of C-O signal after the reduction, observed as well in the analysis of the C1s peak.

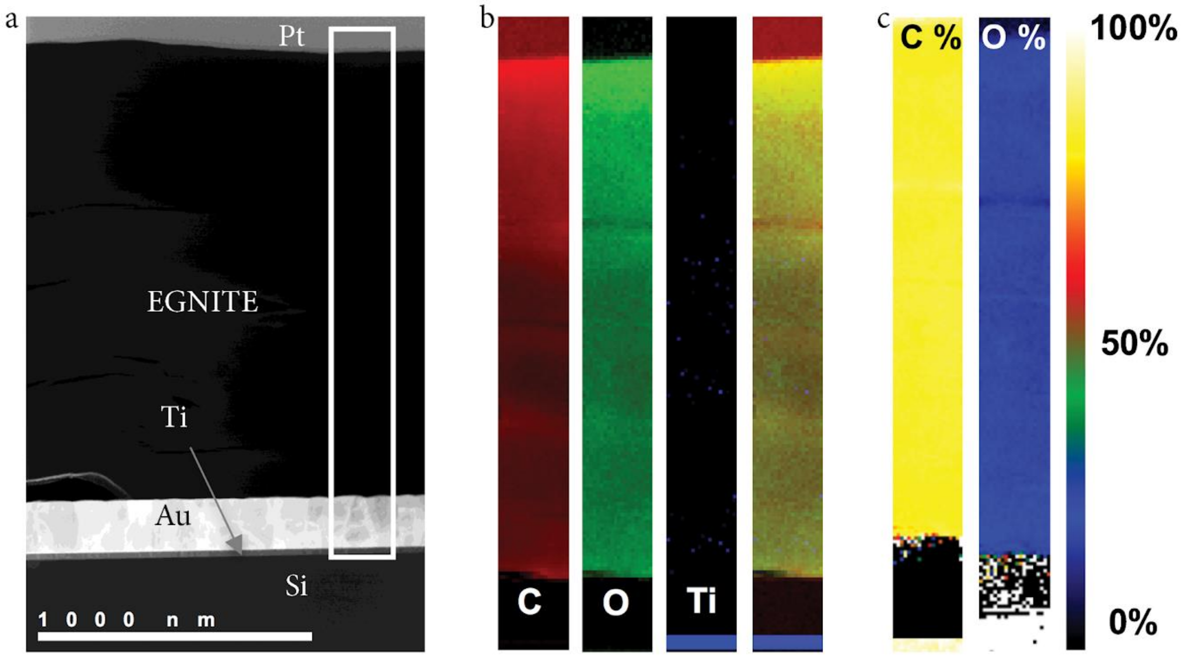

Figure SI 3. Electron energy loss spectroscopy.

a. High-angle annular dark-field (HAADF) scanning transmission electron microscopy (STEM) image of an EGNITE lamella. **b.** STEM Electron energy loss spectroscopy (EELS) elemental maps obtained on the selected area from a. as indicated in the white box by using: C k-edge at 284 eV (red), O K-edge at 532 eV (green) and Ti L-edge at 456 eV (blue), as well as composite of C-O-Ti. **c.** Relative atomic composition for C and O. Carbon is present in almost 85% whereas oxygen reaches 15%.

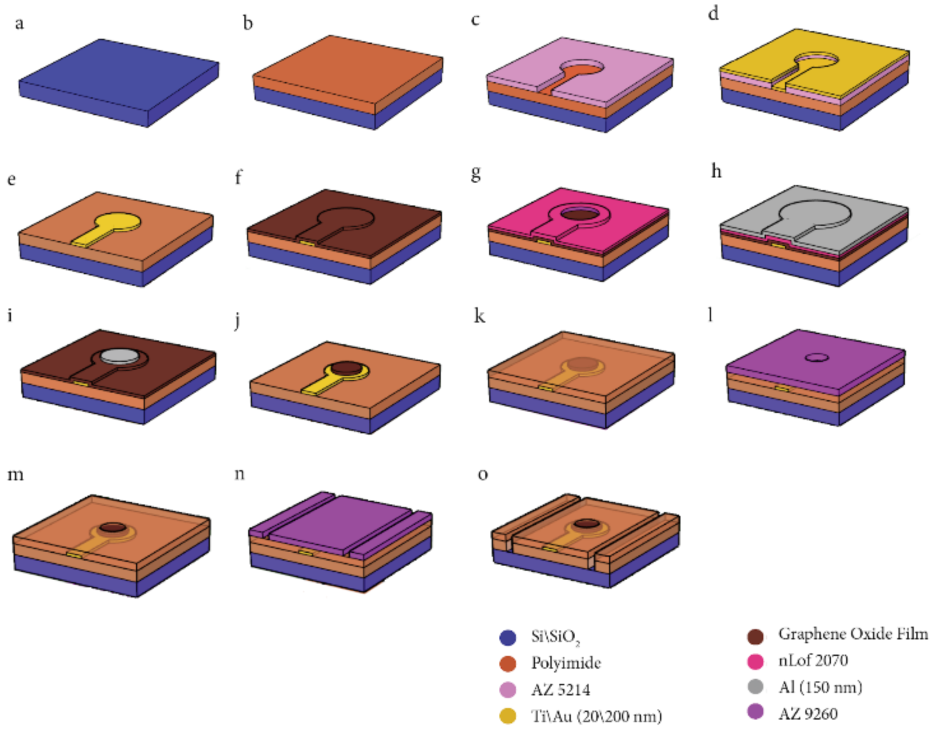

Figure SI 4. Fabrication steps

a. Devices were fabricated on 4" Si/SiO_2 (400 µm / 1 µm) wafers. **b.** First, a 10 µm thick layer of polyimide (PI-2611, HD MicroSystems) was spin coated on the wafer and baked in an atmosphere rich in nitrogen at 350 ºC for 30 minutes. **c-e.** Metallic traces were patterned using optical lithography of the image reversal photoresist (AZ5214, Microchemicals GmbH, Germany). Electron-beam evaporation was used to deposit 20 nm of Ti and 200 of Au and lift-off was performed. **f-j.** After transferring the GO film, aluminum was e-beam evaporated and areas on top of the future microelectrodes were defined by using a negative photoresist (nLOF 2070, Microchemicals GmbH, Germany) and lift off. **j.** Next, the GO film was etched everywhere apart from the future microelectrodes using an oxygen reactive ion etching (RIE) for 5 minutes at 500 W and the protecting Al columns were etched with a diluted solution of phosphoric and nitric acids. **k.** After, a 3 µm thick layer of PI-2611 was deposited onto the wafer and baked as previously described. **l-m.** PI-2611 openings on the microelectrode were then defined using a positive thick photoresist (AZ9260, Microchemicals GmbH, Germany) that acted as a mask for a subsequent oxygen RIE. **n-o.** Later, the devices were patterned on the PI layer using again AZ9260 photoresist and RIE. The photoresist layer was then removed in acetone and the wafer cleaned in isopropyl alcohol and dried out. **p.** Finally, the devices were peeled off from the wafer and were ready to be placed in sterilization pouches to be hydrothermally treated at 134 ºC in a standard autoclave for 3 hours.

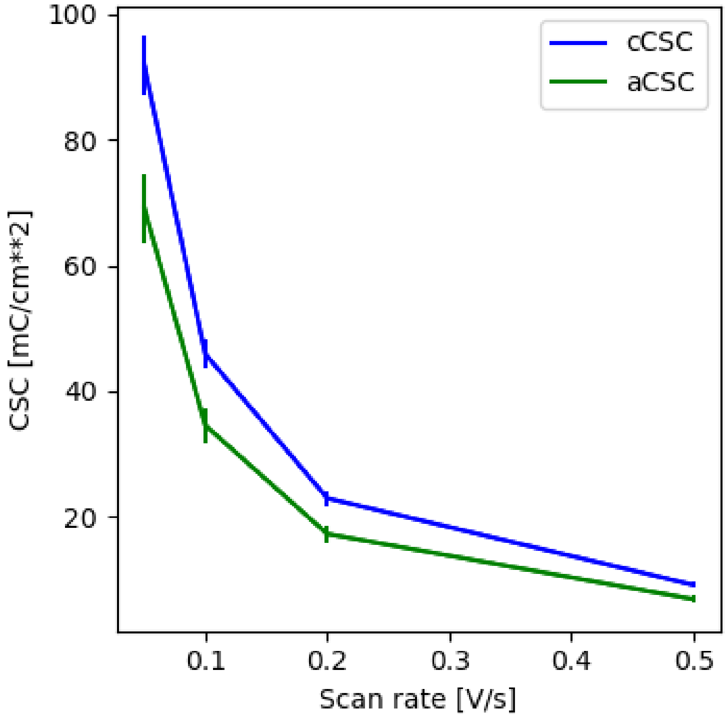

Figure SI 5. CSC vs scan rate
Cathodic charge storage capacity (cCSC, blue) and anodic CSC (aCSC, green) for different scan rates. Data are mean plus minus standard deviation, n=3 electrodes.

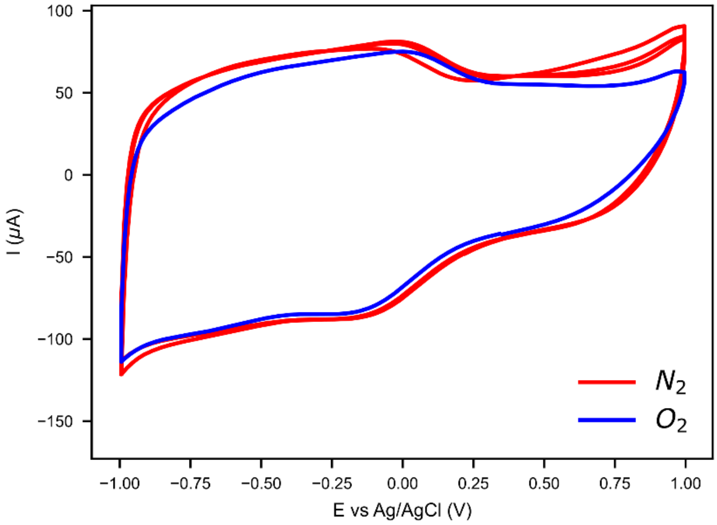

Figure SI 6. Oxygen effect on CV.
Cyclic voltammograms at scan rate of 50 mV/s of an EGNITE electrode in PBS under standard atmosphere conditions (O2, blue line) and under N2-purged electrolyte (red line). No significant differences are observed in the shape of the CV.

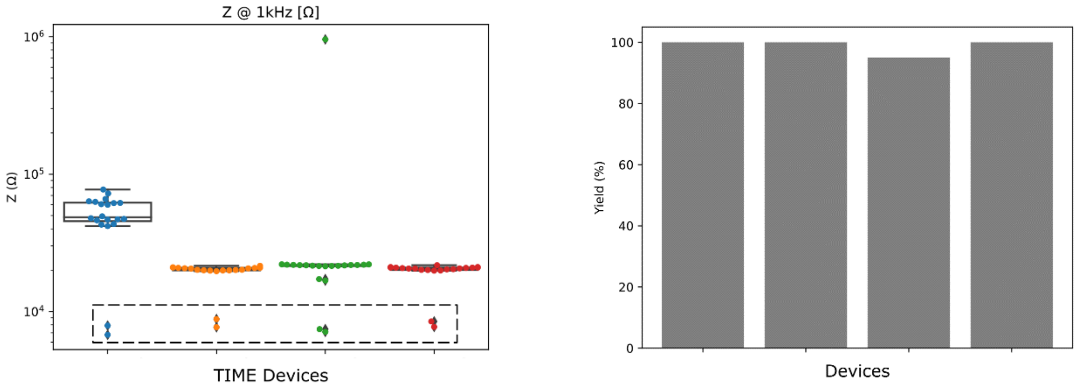

Figure SI 7. Homogeneity and yield of EGNITE microfabrication technology.
Left: Extracted impedance at 1 kHz for different fabricated TIME devices (n=4 with 20 electrodes each) showing high homogeneity. Electrodes within the dashed box with lower impedance correspond to the large rectangular electrodes included in the TIME designs (two in each array). Boxplots: the median, quartile box and minimum and maximum values are presented. Right: Device yield in percentage for each TIME device. Z at 1kHz $<10^5$ Ω has been used as threshold for considering a working electrode.

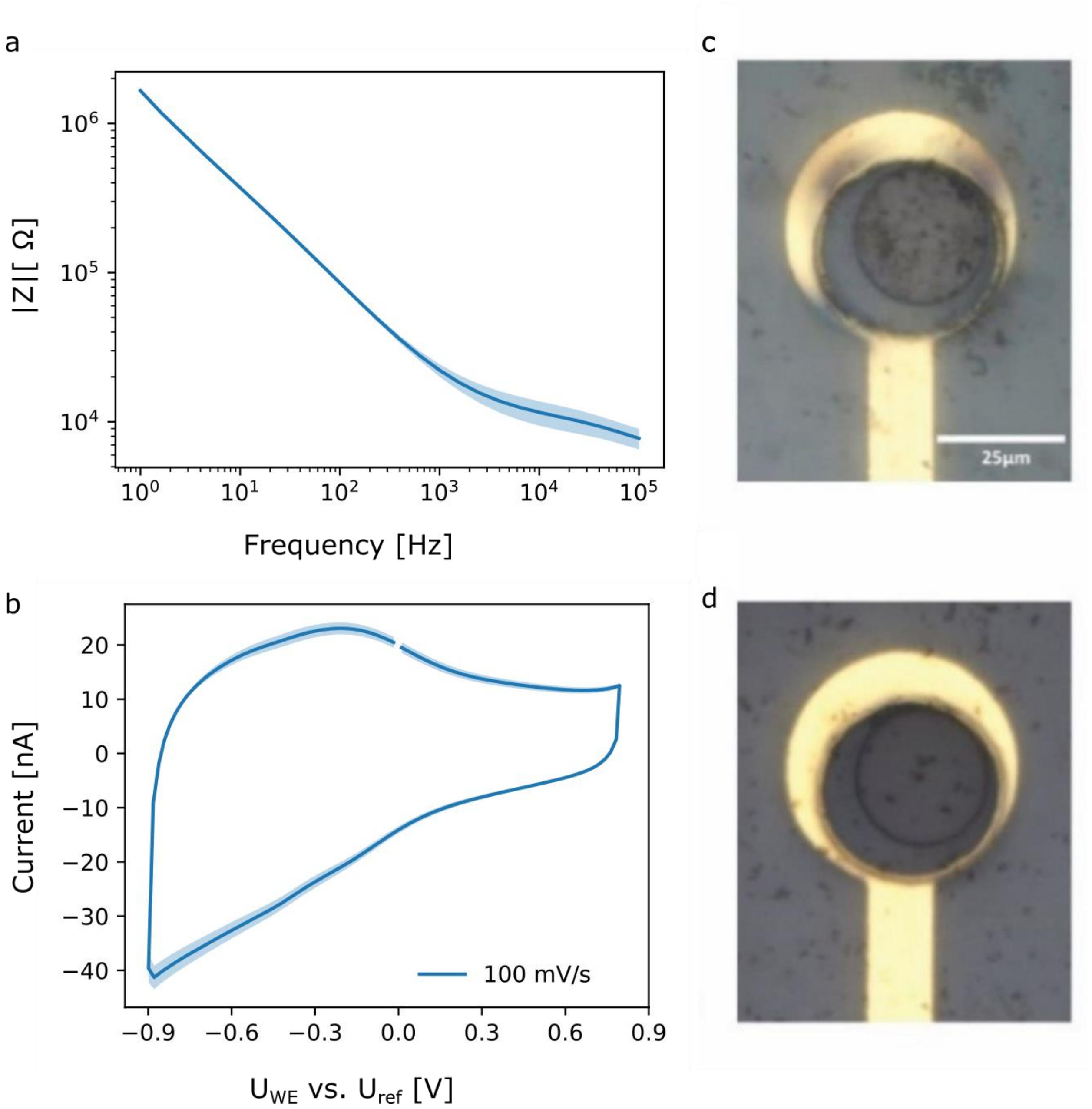

Figure SI 8. EGNITE microelectrodes sustain millions of pulses

a, Impedance spectra and **b,** Cyclic voltammetry (100 mV/s scan rate) measured after 15 million continuous stimulation with 15 µA (3 mC/cm^2) biphasic pulses of 1 ms/phase.Data are mean (solid lines) ± SD (shaded area), n = 3 electrodes.**c-d** Image of 25 µm diameter EGNITE microelectrode before (**g**) and after (**h**) 15 million pulses of continuous stimulation.

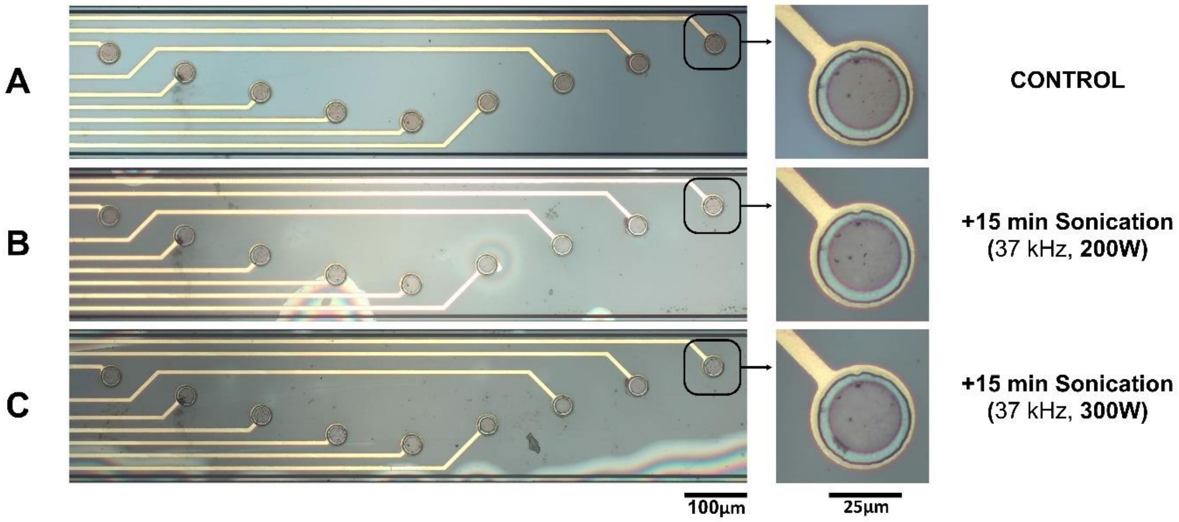

Figure SI 9. Mechanical stability of EGNITE electrodes subjected to sonication in ultrasound bath.

Condition of EGNITE electrodes fabricated in a polyimide device shown **a,** before sonication, **b**, after 15 minutes of sonication at 37 kHz, 200 W, and **c**, after an additional 15 minutes of sonication at 37 kHz, with power elevated to 300 W. Enlarged electrode images are shown to the right of electrode array image.

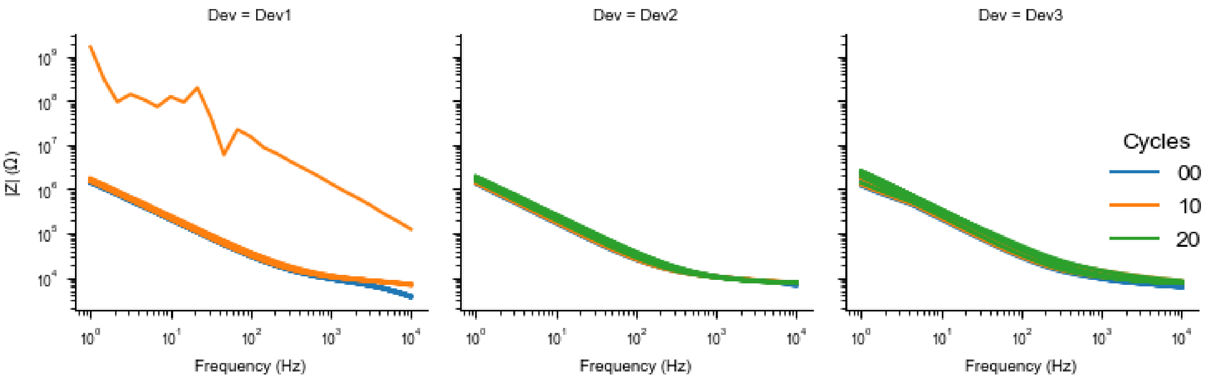

Figure SI 10. Mechanical (Bending) test.

Impedance spectrum for each microelectrode of the three devices that underwent 10 and 20 bending cycles. N=3 devices with 18 microelectrodes each.

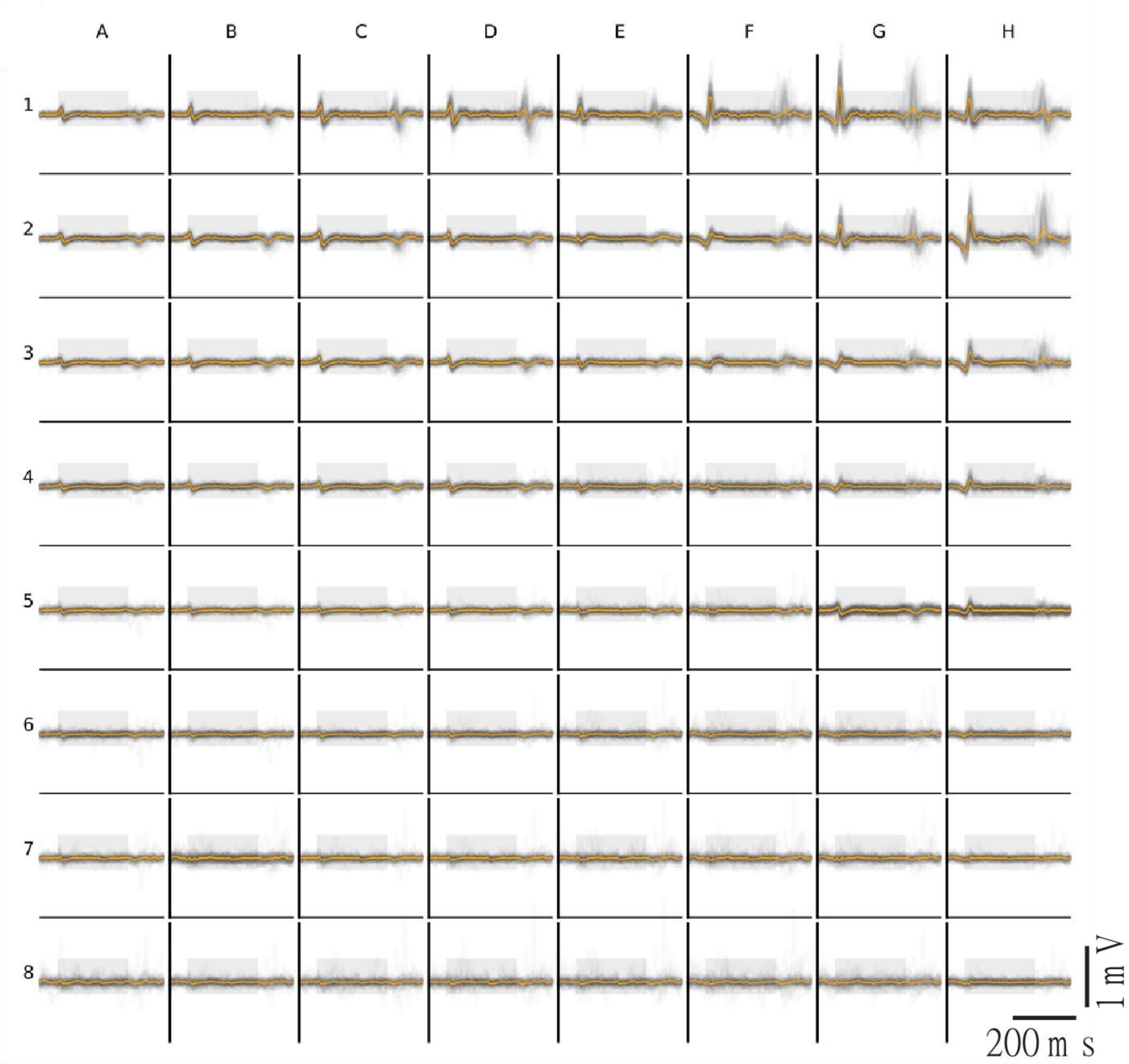

Figure SI 11. Average of neural evoked activity recordings.
Averaged mappings over 20 events corresponding to stimulus of 16 kHz. Each single event is plotted in light grey and the averaged signal in orange.

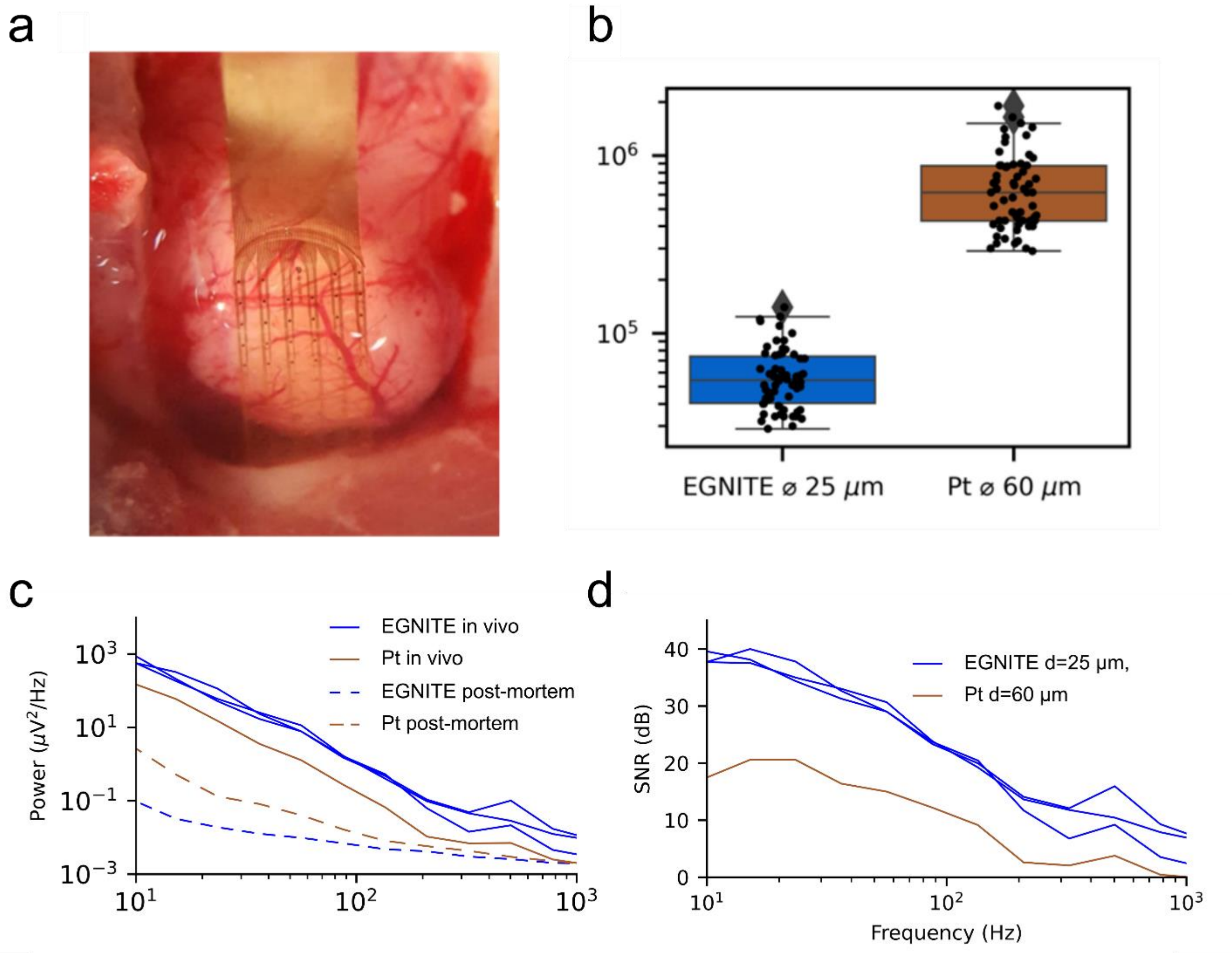

Figure SI 12. Benchmarking of EGNITE vs Pt microelectrodes.

a. Flexible array of 60 Pt microelectrodes of 60 µm diameter (E64-500-20, NeuroNexus, USA) placed on the auditory cortex of a rat. **b.** Impedance measured at 1kHz comparison between an EGNITE array of 64 microelectrodes of 25 µm diameter and a Pt array of 60 microelectrodes of 60 µm diameter. Boxplots: the median, quartile box and minimum and maximum values are presented. **c.** Median power spectral density comparison between EGNITE and Pt microelectrodes measured under in vivo and post-mortem conditions. **d.** Signal-to-noise ratio comparison between 25 µm diameter EGNITE and 60 µm diameter Pt microelectrodes. Data correspond to 192 EGNITE electrodes from n=3 experiments and 60 Pt electrodes from n=1 experiment.

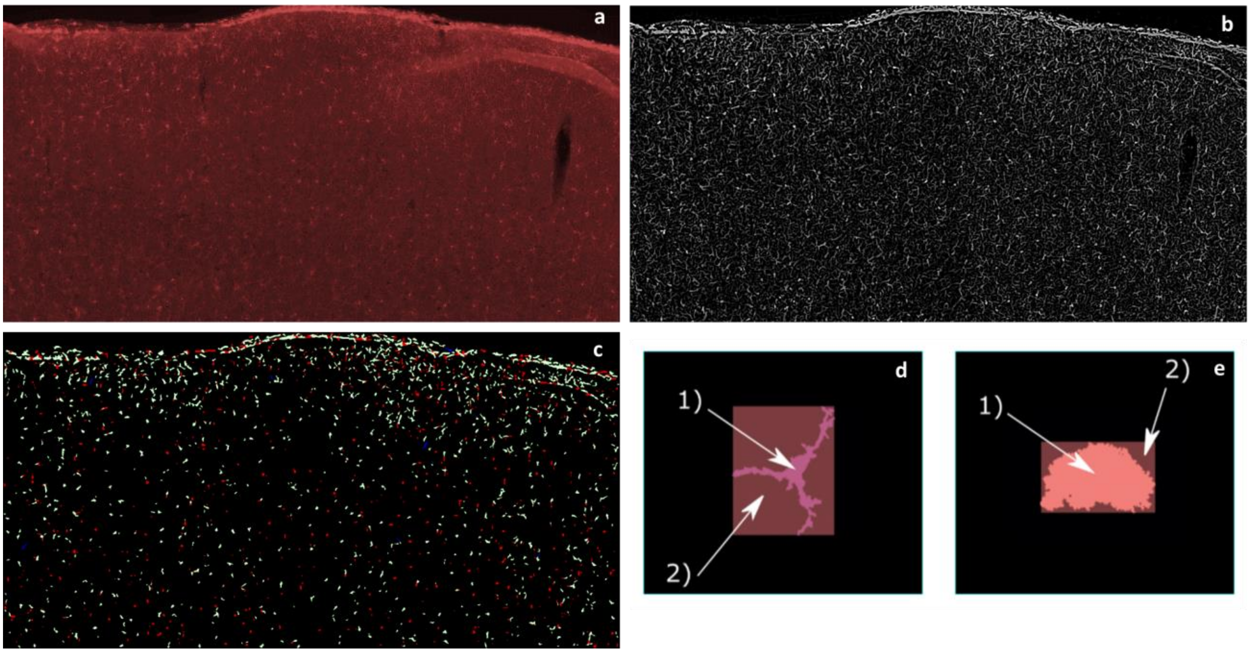

Figure SI 13. Microglia phenotype morphological classification and image analysis. The Classifier (see **Methods**) was run on the 8x magnification images, a representative shown in a. These were enhanced to improve cell segmentation (b). The CellProfiler software was used to classify cells as green - indicating ‘non-activated’ cells; or red cells - classed as ‘activated’ (c). Non-activated microglia (d) have small cell bodies (indicated with #1) and long processes (indicated with #2), so they occupy a smaller proportion of their bounding box than activated microglia (e), which have large cell bodies (#1) and no processes (#2).

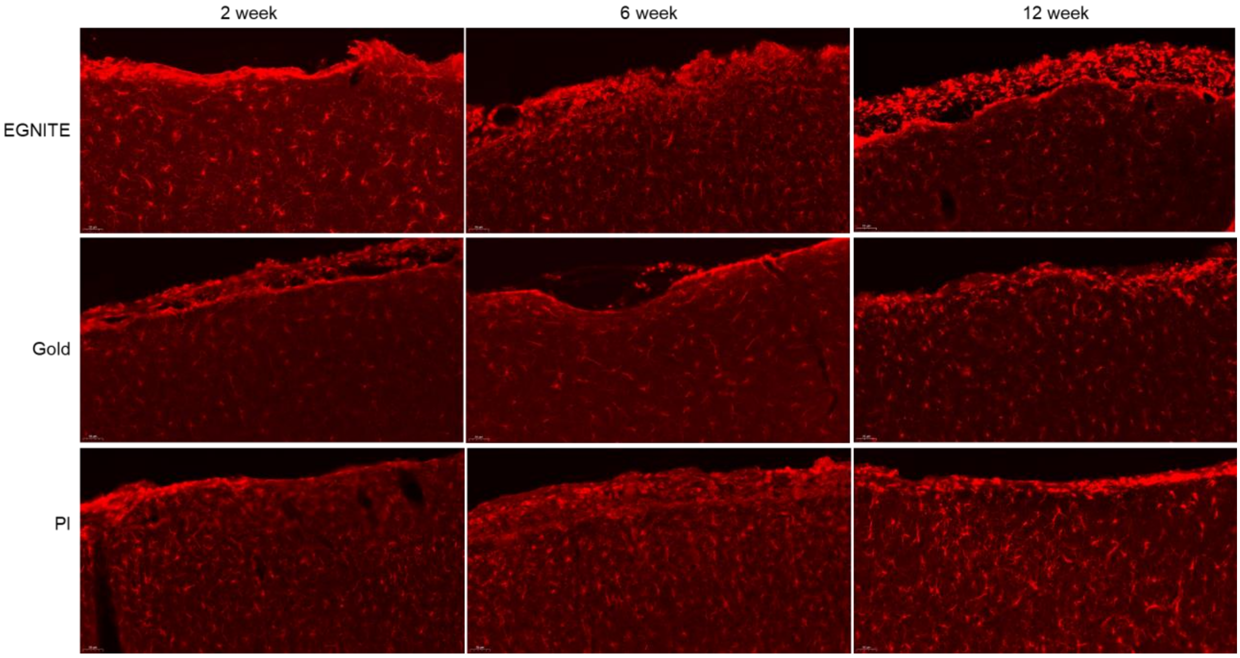

Figure SI 14. Iba-1 Tissue Immunohistochemistry.
Representative images (at 20x magnification, corresponding to Fig.5a yellow box) of cortical tissue sections on direct contact with the implanted microelectrodes consisting of different materials (EGNITE, Gold, Polyimide) and timepoints post-implantation (2, 6 and 12 weeks).

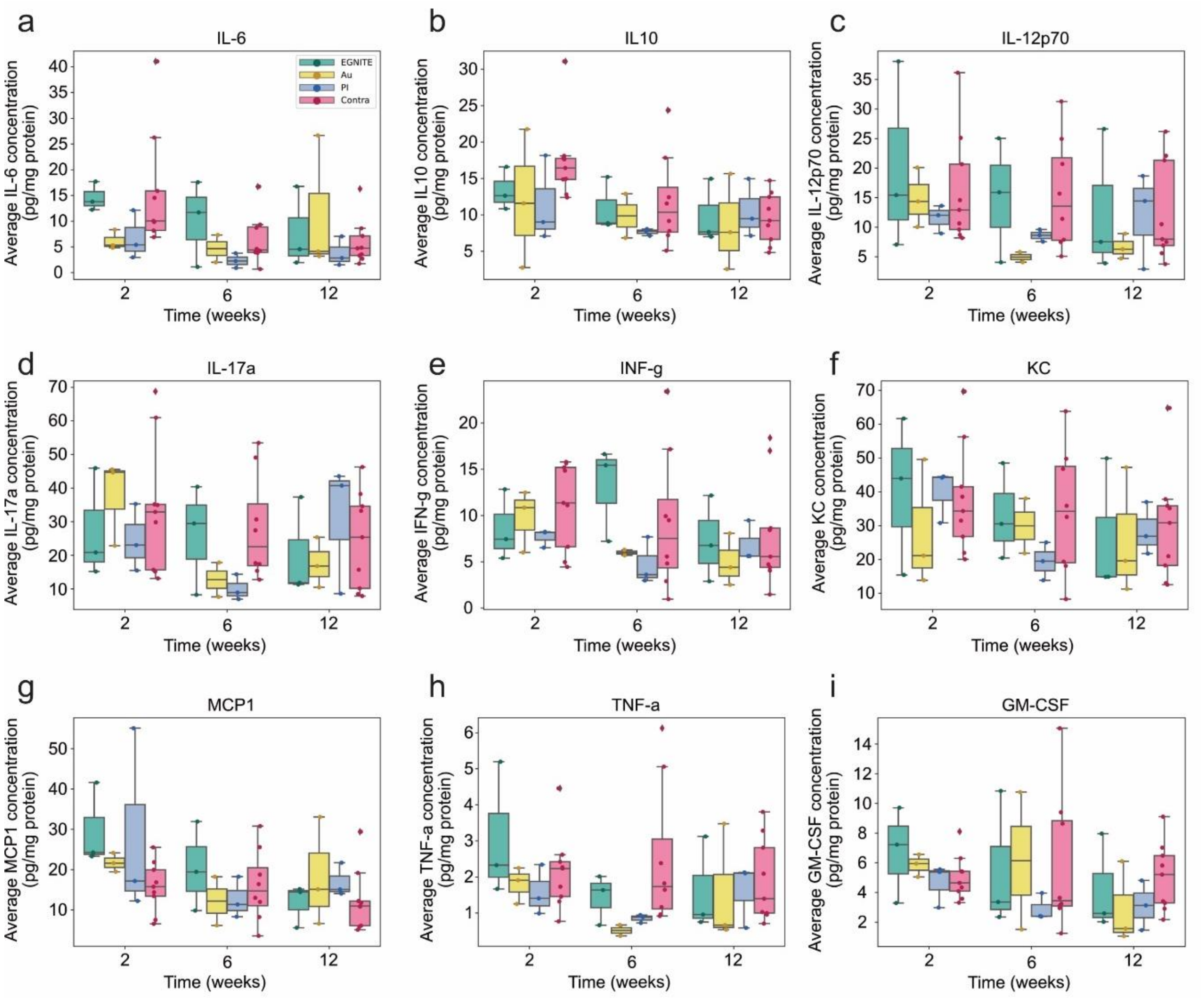

Figure SI 15. Cytokine secretion (by ELISA) following chronic epicortical biocompatibility.

Devices containing EGNITE contacts covering an area of 3 x 3.7 mm were implanted on the cortical surface of the right hemisphere of the rat brain. Quantification of the inflammatory response was assessed over the 12-week period following epicortical implantation for devices with different materials at the passivation opening (EGNITE; Au; and no electrode material, just polyimide: PI). Contra refers to the contralateral hemisphere where no device was implanted. **a-i)** The presence of anti- (IL-10 and IL-18), pro-inflammatory (IL-12p70, IL-17a, IFN-g, MCP1, TNF-a) and inflammatory mediated cytokines (ILI-6, GM-CSF and KC) in the cortical brain tissue were assessed at 2, 6 and 12 weeks post implantation by ELISA using the LEGENDplex™ Rat Inflammation Panel. Cytokine levels were normalised to total protein content (mg/mL) of the tissue sample. EGNITE device implantation did not significantly alter the levels of inflammatory cytokines present in the brain at any timepoint of the experiment. IL-6 and MCP1 levels show a small but non-significant increase at 2 weeks post-implantation with EGNITE, however, by 12 weeks results were analogous to the other device materials tested. Boxplots: the median, quartile box and minimum and maximum values (excluding outliers) are presented. n = 3 for each device, n = 9 for contralateral hemispheres.

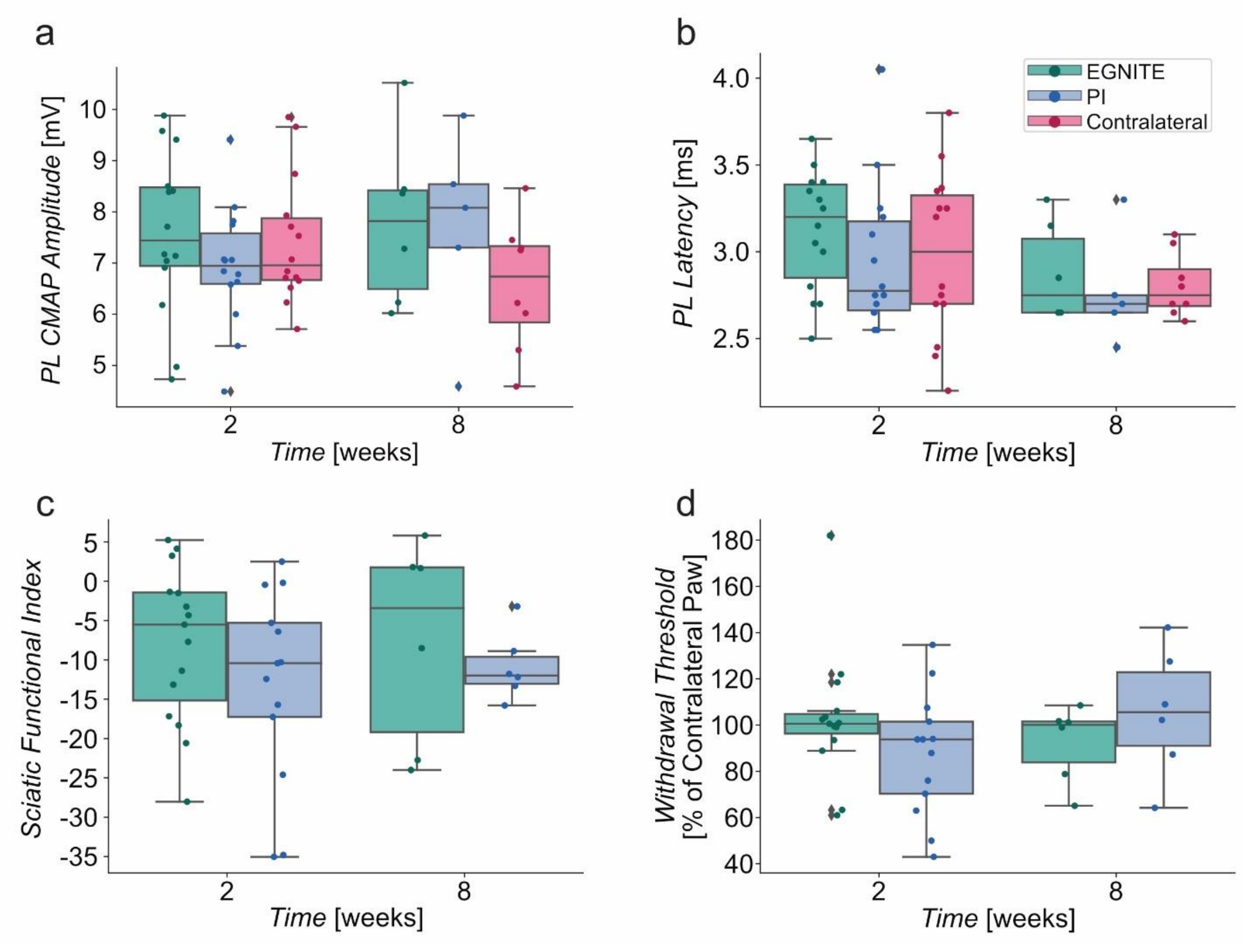

Figure SI 16. Functional assessment after longitudinal nerve implants.

a, b. CMAP amplitude (a) and latency (b) in the PL muscle. **c.** Sciatic functional index obtained in the walking track test for implanted animals with and without EGNITE. Values around ±10 indicate normal values of healthy animals. **d.** Nociceptive evaluation of the hind paw. Results are expressed as percentage of ipsilateral vs contralateral limb. Damage of small nerve fibers would decrease the pain threshold lowering the ratio between the implanted and the intact paw. Boxplots: the median, quartile box and minimum and maximum values (excluding outliers) are presented. N numbers are 13 for the EGNITE group at 2 weeks and 12 for the PI and contralateral, and n=6 for all three groups at 8 weeks.

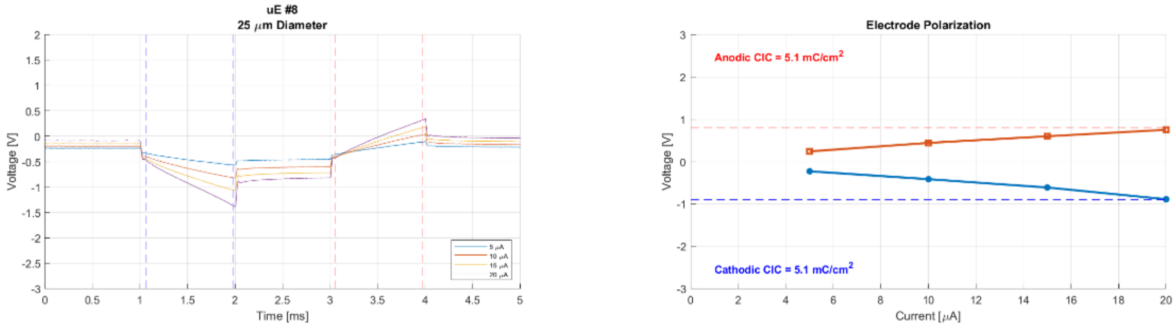

Figure SI 17. Charge-injection limit (CIL) calculation.

Left: Voltage traces of a stimulating electrode at current levels of 5, 10, 15 and 20 µA. Vertical dashed lines indicate the points to get the voltage difference at each the cathodic and anodic phase. Right: Voltage polarization of the electrode for each current pulse amplitude as determined from the vertical lines in the left plot. Horizontal lines show the EGNITE electrochemical window (-0.9 to 0.8 V).
