# Supplementary material for: Nanoporous graphene-based thin-film microelectrodes for in vivo high-resolution neural recording and stimulation
Source: Nat Nanotechnol. 2024 Jan 11;19(4):514–23. doi: 10.1038/s41565-023-01570-5 (PMC11026161; doi:10.1038/s41565-023-01570-5)
Supplement: Supplementary file 2 — Reporting Summary [file 41565_2023_1570_MOESM2_ESM.pdf]

nature portfolio

Corresponding author(s): Jose A. Garrido

Last updated by author(s): Oct 20, 2023

Reporting Summary

Nature Portfolio wishes to improve the reproducibility of the work that we publish. This form provides structure for consistency and transparency in reporting. For further information on Nature Portfolio policies, see our Editorial Policies and the Editorial Policy Checklist.

Statistics

For all statistical analyses, confirm that the following items are present in the figure legend, table legend, main text, or Methods section.

n/a	Confirmed	
☐	☒	The exact sample size (*n*) for each experimental group/condition, given as a discrete number and unit of measurement
☐	☒	A statement on whether measurements were taken from distinct samples or whether the same sample was measured repeatedly
☐	☒	The statistical test(s) used AND whether they are one- or two-sided<br>*Only common tests should be described solely by name; describe more complex techniques in the Methods section.*
☒	☐	A description of all covariates tested
☐	☒	A description of any assumptions or corrections, such as tests of normality and adjustment for multiple comparisons
☐	☒	A full description of the statistical parameters including central tendency (e.g. means) or other basic estimates (e.g. regression coefficient) AND variation (e.g. standard deviation) or associated estimates of uncertainty (e.g. confidence intervals)
☐	☒	For null hypothesis testing, the test statistic (e.g. *F*, *t*, *r*) with confidence intervals, effect sizes, degrees of freedom and *P* value noted<br>*Give P values as exact values whenever suitable.*
☒	☐	For Bayesian analysis, information on the choice of priors and Markov chain Monte Carlo settings
☒	☐	For hierarchical and complex designs, identification of the appropriate level for tests and full reporting of outcomes
☒	☐	Estimates of effect sizes (e.g. Cohen's *d*, Pearson's *r*), indicating how they were calculated

Our web collection on statistics for biologists contains articles on many of the points above.

Software and code

Policy information about availability of computer code

Data collection	Standard procedures were used for data collection as especified in the main text.
Data analysis	No custom code was used for data collection. Custom code developed for neurophysiological analysis is available at https://github.com/aguimera/PhyREC.

For manuscripts utilizing custom algorithms or software that are central to the research but not yet described in published literature, software must be made available to editors and reviewers. We strongly encourage code deposition in a community repository (e.g. GitHub). See the Nature Portfolio guidelines for submitting code & software for further information.

Data

Policy information about availability of data

All manuscripts must include a data availability statement. This statement should provide the following information, where applicable:

- Accession codes, unique identifiers, or web links for publicly available datasets
- A description of any restrictions on data availability
- For clinical datasets or third party data, please ensure that the statement adheres to our policy

The experimental data that support the figures within this paper and other findings of this study can be accessed by contacting the corresponding author. Authors can make data available on request, agreeing on data formats needed.

Research involving human participants, their data, or biological material

Policy information about studies with human participants or human data. See also policy information about sex, gender (identity/presentation), and sexual orientation and race, ethnicity and racism.

Reporting on sex and gender	No human data was used in this project.
Reporting on race, ethnicity, or other socially relevant groupings	No human data was used in this project.
Population characteristics	No human data was used in this project.
Recruitment	No human data was used in this project.
Ethics oversight	No human data was used in this project.

Note that full information on the approval of the study protocol must also be provided in the manuscript.

Field-specific reporting

Please select the one below that is the best fit for your research. If you are not sure, read the appropriate sections before making your selection.

☒ Life sciences ☐ Behavioural & social sciences ☐ Ecological, evolutionary & environmental sciences

For a reference copy of the document with all sections, see nature.com/documents/nr-reporting-summary-flat.pdf

Life sciences study design

All studies must disclose on these points even when the disclosure is negative.

Sample size	Sprague Dawley rats were used in this study. No sample-size calculation was performed. Sample size was sufficient for proof-of-concept of EGNITE technology. For the cortical biocompatibility studies where some prior knowledge about variability was available, we performed a power calculation indicating that groups of 3-4 animals at each time point would be required to sufficiently power these sets of experiments. For cortical histological staining, all data sets are n=3. For intraneural biocompatibility studies datasets are n=6-8.
Data exclusions	The exact sample size and whether data is obtained from the same or multiple subjects is mentioned in the text. Otherwise, data was selected for illustrative purposes of EGNITE capabilities.
Replication	All major findings were replicated (see sample size on figure captions), and the results reproducible. Experimental results were very comparable across experimental units (animals), and electrodes. Single proof-of-concept experiments are also included as demonstration of technological capabilities.
Randomization	Randomization is not rellevant in our study since no conclusion is based on subject-dependent effects.
Blinding	All post-hoc biocompatibility analysis was performed by a researcher blind to the experimental condition. Electrophysiology data analysis did not require blinding as all animals were treated in the same way.

Reporting for specific materials, systems and methods

We require information from authors about some types of materials, experimental systems and methods used in many studies. Here, indicate whether each material, system or method listed is relevant to your study. If you are not sure if a list item applies to your research, read the appropriate section before selecting a response.

Materials & experimental systems

n/a	Involved in the study	
☐	☒	Antibodies
☒	☐	Eukaryotic cell lines
☒	☐	Palaeontology and archaeology
☐	☒	Animals and other organisms
☒	☐	Clinical data
☒	☐	Dual use research of concern
☒	☐	Plants

Methods

n/a	Involved in the study	
☒	☐	ChIP-seq
☒	☐	Flow cytometry
☒	☐	MRI-based neuroimaging

Antibodies

Antibodies used	Intreneural: Primary antibodies: for myelinated axons (RT97 to label Neurofilament 200K, 1:200, Developmental Studies Hybridoma Bank, DSHB Cat# rt97, RRID:AB_528399) and macrophages (iba1, 1:500, Wako, 19-19741). Secondary antibodies donkey anti-Mouse Alexa fluor 488 (A21202) and donkey anti-Rabbit Alexa fluor 555 (Invitrogen, 1:200, Ab150070). Cortical: primary antibody anti-Iba-1 (1:1000, 019-19741, Wako). Secondary antibody, anti-rabbit Alexa Fluor 594 (A-11012, 1:400; Thermofisher).
Validation	Anti-Iba-1 (Wako 019-19741). This antibody is confirmed by the manufacturer to react with rat IBA1 and validation images can be obtained here: https://labchem-wako.fujifilm.com/us/product/detail/W01W0101-1974.html<br><br>RT97: According to on the manufacturer's website:<br>Confirmed Species Reactivity: Bovine, Chicken, Human, Mouse, Rat<br>Additional Information: RT97 reacts with neurofibrillary tangles and plaques [PMID 6178036]. RT97 cross-reacts with neurofilament medium. RT97 has been used as a cell marker for neurons.<br>Relevant citation provided on the manufacturer's website:<br>Investigative ophthalmology & visual science 51.4 (2010 Apr): 2248-62.<br>Pentoxifylline promotes recovery of erectile function in a rat model of postprostatectomy erectile dysfunction.<br>Lue TF<br>Other relevant citations:<br>[1] I. Delgado-Martínez, M. Righi, D. Santos, A. Cutrone, S. Bossi, S. D?Amico, J. Del Valle, S. Micera, X. Navarro, S. D'Amico, Fascicular nerve stimulation and recording using a novel double-aisle regenerative electrode, J. Neural Eng. 14 (2017) 046003. https://doi.org/10.1088/1741-2552/aa6bac.<br><br>Iba1: According to on the manufacturer's website:<br>Confirmed Species Reactivity: Human, Mouse, Rat,<br>Additional Information: Iba1 (Ionized calcium-binding adapter molecule1) is an approximately 17 kDa calcium-binding protein. It is used as a microglial marker because it is expressed specifically in microglia in the central nervous system1). It is expressed in both resting and activated microglia, but is reportedly expressed more highly in activated microglia2). It is also expressed in macrophages in peripheral tissues and is known as AIF-1 (Allograft inflammatory factor-1). Iba1 binds to F-actin in cells to form actin bundles. The formation of actin bundles is thought to be required for the membrane ruffling observed during cell migration and phagocytosis3).<br>Relevant citation provided on the manufacturer's website:<br>Sasaki, Y., Ohsawa, K., Kanazawa, H., Kohsaka, S., & Imai, Y. Biochem. Biophys. Res. Commun., 286(2), 292(2001).<br>Iba1 is an actin-cross-linking protein in macrophages/microglia.<br>Other relevant citations:<br>[1] N. De la Oliva, X. Navarro, J. del Valle, Dexamethasone Reduces the Foreign Body Reaction to Intraneural Electrode Implants in the Peripheral Nerve of the Rat, Anat. Rec. 301 (2018) 1722–1733. https://doi.org/10.1002/ar.23920.<br><br>Iba-1 (Wako 019-19741): IHC in rats is included https://labchem-wako.fujifilm.com/us/product_data/docs/00055446_doc02.pdf

Animals and other research organisms

Policy information about studies involving animals; ARRIVE guidelines recommended for reporting animal research, and Sex and Gender in Research

Laboratory animals	Neural recording: Sprague Dawley rats, males, 4-months old.<br>Chronic Epicortical Biocompatibility: Adult (6-7 weeks old), male, Sprague-Dawley rats(Charles River, England).<br>Intraneural Stimulation and biocompatibility: Female Sprague-Dawley rats (250-300 g, ~18 weeks old).
Wild animals	No wild animals were used in this study.
Reporting on sex	Not aplicable in this study.
Field-collected samples	No field-collected samples were used in this study.
Ethics oversight	Neural recording: All experimental procedures were performed in accordance with the recommendations of the European Community Council and French legislation for care and use of laboratory animals. The protocols were approved by the Grenoble ethical committee (ComEth) and authorized by the French ministry (number 04815.02).<br>Chronic Epicortical Biocompatibility: Experimental procedures were carried out in accordance with the Animals (Scientific Procedures) Act 1986, under the approval of the Home Office and University of Manchester local animal welfare ethical review body (AWERB) (Project Licence P089E2EOA).<br>Intraneural Stimulation and biocompatibility: All animal experiments were approved by the Ethical Committee of the Universitat Autònoma de Barcelona in accordance with the European Communities Council Directive 2010/63/EU.

Note that full information on the approval of the study protocol must also be provided in the manuscript.
